# Tick lipocalin triggers mammalian IGFBP-3-mediated apoptosis in macrophages and keratinocytes

**DOI:** 10.3389/fimmu.2026.1768484

**Published:** 2026-02-18

**Authors:** Krittika Nandy, P. P. Mahesh, Lichao Liu, Daniel E. Sonenshine, Hameeda Sultana, Girish Neelakanta

**Affiliations:** 1 Department of Biomedical and Diagnostic Sciences, College of Veterinary Medicine, University of Tennessee, Knoxville, TN, United States; 2 Department of Biological Sciences, Old Dominion University, Norfolk, VA, United States; 3 Vector Molecular Biology Section, Laboratory of Malaria and Vector Research, National Institute of Allergy and Infectious Diseases, National Institutes of Health, Rockville, MD, United States

**Keywords:** anti-apoptotic, apoptosis, Bcl-2, caspases, ticks, cytokines, IGFBP-3, lipocalin

## Abstract

Ticks secrete several molecules, including lipocalins, in their saliva during blood feeding on a vertebrate host. In this study, we provide novel evidence on the role of *Ornithodoros turicata americanus* tick lipocalin (Otlip) in modulating cytokine expression and in triggering apoptosis in mammalian macrophages and keratinocytes. The cytokine array analysis revealed significantly increased secretion of insulin-like growth factor-binding protein 3 (IGFBP3) from murine macrophage cell line upon treatment with recombinant Otlip protein (rGST-Otlip) when compared to the secretion noted from cells treated with a control protein (rGST). Similar observation with cytokine protein array analysis was noted when murine macrophages were treated with salivary gland lysates generated from fed *O. turicata americanus* ticks. In addition, we noted increased expression of IGFBP3 in human keratinocytes cell line upon treatment with rGST-Otlip. The Live/Dead staining and TUNEL microscopic analysis revealed that treatment with rGST-Otlip induced apoptotic cell death in murine macrophage and human keratinocytes cell lines. qRT-PCR analysis showed increased *caspase-3* and reduced *bcl-2* (an anti-apoptotic protein) transcripts in both murine macrophages and human keratinocytes cell lines upon treatment with rGST-Otlip. Immunoblotting further showed increased Caspase-3 levels in both cells lines upon treatment with rOtlip. Furthermore, our study noted that siRNA-mediated silencing of *igfbp3* (carrier protein for IGF) expression inhibited rGST-Otlip-mediated apoptosis in murine macrophages. Taken together, our study not only provides new insights into the role of arthropod salivary molecules in the interactions with mammalian cells but also could lead to the development of strategies to target tick blood feeding.

## Introduction

Ticks are hematophagous arthropods that transmit several pathogens to humans and animals (1–3). To overcome host defenses during blood feeding, ticks secrete saliva that contains a plethora of bioactive molecules including anticoagulants (4–7), hemostasis and immunity suppressors, anti-platelet aggregation factors (8), anti-vasodilatory molecules (4, 5, 8), T-cell activation inhibitors (4, 5, 8, 9), anti-wound healing (10, 11), and anti-complement factors. For instance, during tick blood feeding, the host releases histamine and serotonin (5-HT) at the bite site to increase vascular permeability and attract recruitment of immune cells (2, 5, 8, 12). This would result in inflammation that causes pain and itching at the bite site and could lead to tick rejection from the host (4–6, 8). However, ticks secrete salivary molecules such as lipocalins that binds to histamines and serotonins to reduce the host immune response during blood feeding (13–15).

Lipocalins are low molecular weight molecules (13, 16). Studies have identified lipocalins from various ticks (13–15, 17). Lipocalins have wide functional diversity with less identity in the amino acid sequences (13–16). However, their three-dimensional structures are conserved (13–16). Lipocalins are classified as core lipocalins and outliers (13, 16–19). Those lipocalins that are conserved in their structure are classified under core lipocalins (13, 16–19). The divergent group of lipocalins from the core group are classified as outliers (13, 16–19). In addition, some of the lipocalins have conserved biogenic amine-binding (BAB) motif (CD[VIL]X_7-17_EL[WY]X_3-30_C) (15). This motif is noted to be important for lipocalin binding to histamine/serotonin molecules (15).

Both hard and soft ticks secrete lipocalins in their saliva during blood feeding (13–15). Hard ticks feed on a vertebrate host for three to 10 days to take a complete blood meal (1, 20). However, soft ticks such as *Ornithodoros turicata*, the vector for relapsing fever spirochete *Borrelia turicatae*, take a complete blood meal between 45 min to one hour (1, 20). Relapsing fever is now considered as one of the neglected tick-borne diseases (21). The life cycle of *O. turicata* involves eggs, larvae, up to seven nymphal stages, and adults (1, 20). Larvae, nymphs, and adult ticks require a blood meal from multiple new hosts to molt into next stage or to lay eggs (1, 20). In the nymphal stages, these ticks also feed on multiple new hosts to molt from one instar to up to seven nymphal instar stages (1, 20). *Ornithodoros turicata* ticks can survive in nature for several years (1, 20). We have previously identified and characterized lipocalin-like molecule (Otlip) from *O. turicata americanus* soft ticks (22). We noted that recombinant Otlip (rGST-Otlip) could bind to histamine in a dose dependent manner (22). In addition, we noted increased expression of *otlip* transcripts in salivary glands compared to expression noted in guts of *O. turicata* americanus ticks (22).

Lipocalins are also now known to perform other functions. They are involved in the inhibition of platelet aggregation and the complement cascade (23). The lipocalin Coversin (rEV576, a recombinant lipocalin protein) from *Ornithodoros moubata* is undergoing clinical trials for treating thrombotic microangiopathy (24). The Japanin (lipocalin molecule) from *Rhipicephalus appendiculatus* ticks influences T cell responses (25). Additionally, tick lipocalins like Ha24 from *Hyalomma asiaticum* and rEV131 and rEV504 from *R. appendiculatus* have shown promise in reducing inflammation and immune responses in disease models (26). These findings highlight the potential role for tick lipocalins as therapeutic agents targeting hemostasis, complement activation, inflammation, and acquired immunity. Furthermore, studies have shown that mammalian lipocalin 2 (LCN2) has important roles in inducing cell death and/or survival (27–29). In addition, mammalian lipocalin is implicated in apoptosis due to interleukin-3 (IL-3) deprivation and iron transport and upon treatment with 13-cis retinoic acid (27, 28). However, very little is known about the role of tick lipocalin in modulating host cell death/apoptosis or survival. In this study, we performed experiments with rGST-Otlip and murine macrophages and human keratinocytic cell lines to understand whether tick lipocalin modulates cytokine responses, survival and/or cell death/apoptosis in these cells.

## Results

### *Ornithodoros turicata americanus* recombinant lipocalin and salivary proteins modulate secretion of cytokines and chemokines from murine RAW macrophages

**Figure 1:**
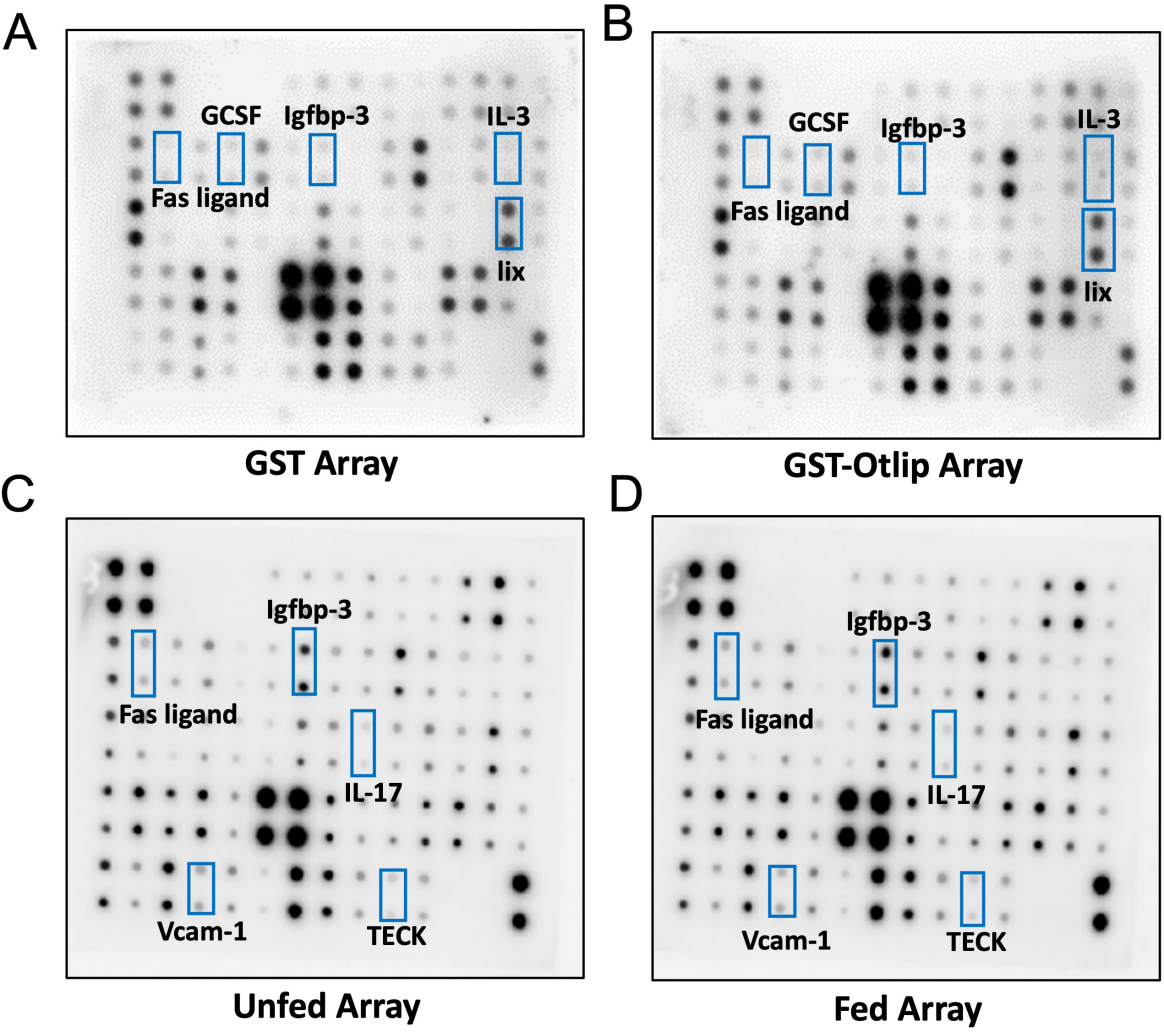
Cytokine array analysis with supernatants obtained from murine macrophages treated with rGST or rGSTOtlip or with salivary gland lysates generated from unfed or fed *O. turicata americanus* ticks. Cytokine profile in the supernatants of murine RAW macrophages treated with either 5µg/mL of rGST **(A)** or rGST-Otlip **(B)** purified proteins or with 5µg/mL salivary gland lysates generated from unfed **(C)** or fed **(D)** ticks is shown. Black spots indicate intensity of cytokine expression. The black boxes indicate the cytokines selected for validation by qRT-PCR and/or immunoblotting analysis.

To examine whether Otlip or any other *O. turicata americanus* salivary factors impact mammalian cytokine and chemokine expression, murine RAW macrophages were treated with recombinant GST (rGST) or GST-Otlip (rGST-Otlip) proteins (**Supplementary Figure 1**) or with lysates prepared from salivary glands isolated from unfed or fed nymphal ticks. RayBiotech cytokine protein array C3 kit was used in these assays (**Supplementary Figure 2**). Membranes that were spotted with antibodies against 62 different cytokines and chemokines were treated with supernatants collected from different treatment groups (**Figure 1**). One membrane was used per treatment group (**Figures 1A–D**). We detected differential signal intensities for the cytokines and chemokines after ~30 to 60 seconds exposure in all treatment groups (**Figure 1**). Some signal intensities were noted to be higher, and some were noted to be lower when different groups were compared (comparison was made between rGST or rGST-Otlip treatment groups or between unfed or fed salivary gland lysate treatment groups). The signal intensity data was collected from with Image-J software. The average of the duplicate signal intensities was calculated; the background was subtracted and normalized to the positive controls.

**Figure 2:**
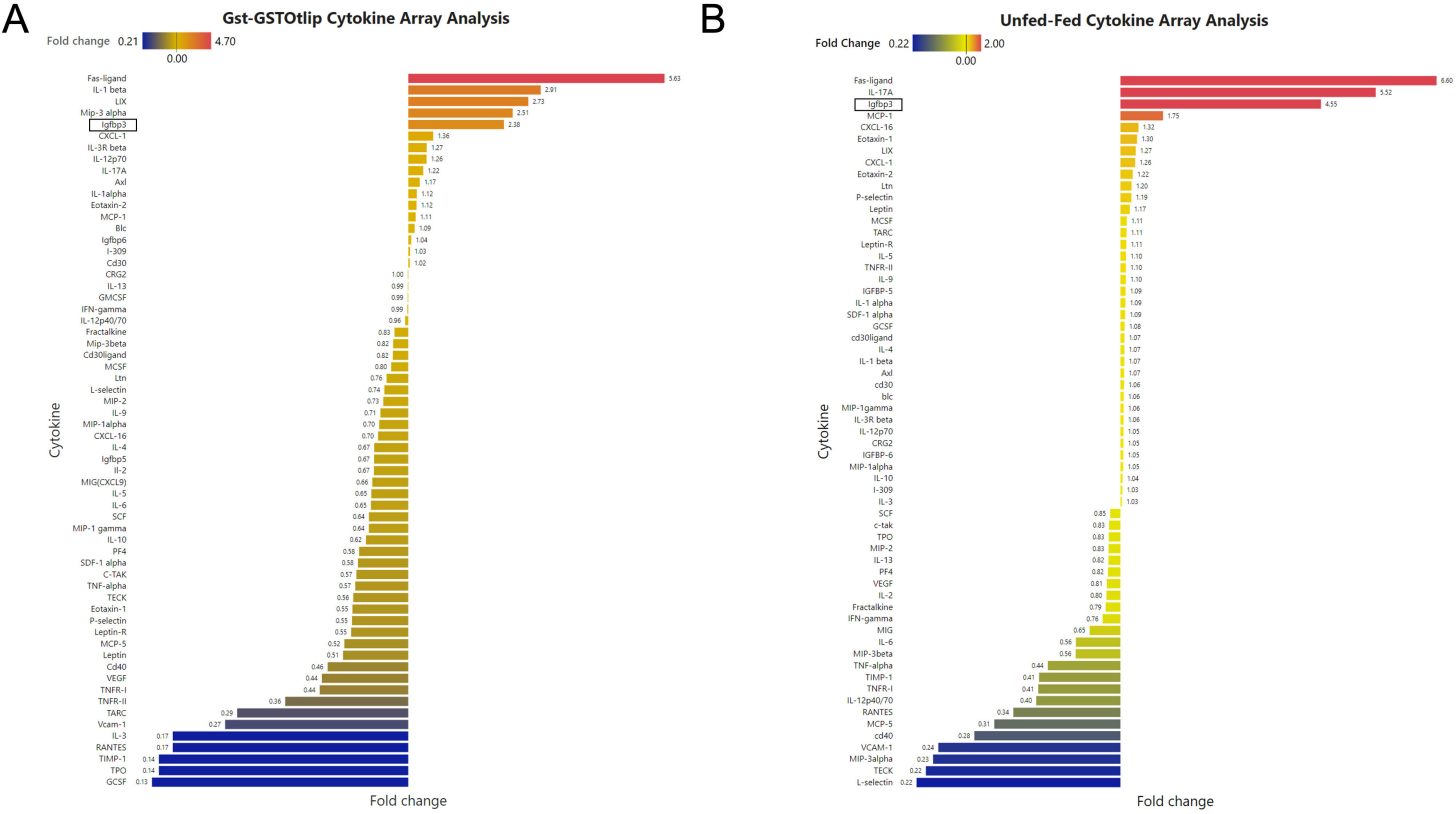
Quantification of cytokine array analysis performed with supernatants from murine macrophages treated with rGST or rGST-Otlip or salivary gland lysates generated from unfed or fed ticks. The bar graphs depicting the differential levels (as a fold change) of murine cytokines/chemokines in supernatants collected from murine macrophages treated with rGST-Otlip relative to the levels noted upon treatment with rGST **(A)** or treated with fed tick salivary gland lysate relative to the levels noted upon treatment with unfed tick salivary gland lysates **(B)**. The cytokine/chemokines with the highest level in the fold change is at the top of the bar graph while the lowest level in the fold change is at the bottom of the bar graph. A fold change value of 1 indicates no change in the expression levels. The graphs were generated using Power-BI software. Protein names are indicated on the Y-axis. IGFBP3 protein is boxed in both arrays.

We then used Power BI software to quantify the levels of secreted cytokine and chemokine from murine macrophages treated with rGST/rGST-Otlip or unfed/fed salivary gland lysates. The cytokines with a Fold change value of 1 were considered having no differential expression. The bar graphs are arranged in a gradient showing the cytokine with the increased fold change at the top and gradually ending in the cytokine with the decreased fold-change at the bottom. The top five cytokines shown in the bar graph that were secreted from RAW macrophages at increased levels upon rGST-Otlip treatment when compared to the levels noted upon rGST treatment are FAS-ligand (5.6 fold), IL-1 beta (2.9 fold), LIX (2.7 fold), MIP-3 alpha (2.5 fold) and IGFBP3 (2.3 fold) (**Figure 2A**). The bottom five cytokines in the bar graph that were secreted at the decreased levels upon rGST-Otlip treatment when compared to levels noted upon GST treatment are GCSF (7.6 fold), TPO (7.1 fold), TIMP-1 (7.1 fold), RANTES (5.8 fold) and IL-3 (5.8 fold) (**Figure 2A**). Similar analysis was performed with data generated from treatment with unfed or fed tick salivary gland lysates. We noted increased levels of FAS-ligand (6.6 fold), IL-17A (5.5 fold), IGFBP3 (4.5 fold), MCP-1 (1.7 fold) and CXCL-16 (1.3 fold) upon treatment with fed tick salivary gland lysates compared to the levels noted upon treatment with unfed salivary gland lysates (**Figure 2B**). In addition, we noted decreased levels of L-selectin (4.5 fold), TECK (4.5 fold), MIP-3 alpha (4.3 fold), VCAM-1 (4.1 fold) and CD40 (3.5 fold) upon treatment with fed tick salivary gland lysates compared to the levels noted upon treatment with unfed tick salivary gland lysates (**Figure 2B**). We noted disturbed cell morphology (rounding up of cells) upon treatment with rGST-Otlip or fed tick salivary gland lysate compared to the morphology noted upon treatment with rGST or unfed tick salivary gland lysate, respectively (**Supplementary Figure 3**). Collectively, these results show that FAS-ligand and IGFBP3 were among the top 5 that are secreted from murine macrophages at increased levels upon treatment with rOtlip or with fed tick salivary gland lysates.

### Treatment with rGST-Otlip and fed tick salivary gland lysates modulate cytokine gene expression in murine macrophages

**Figure 3:**
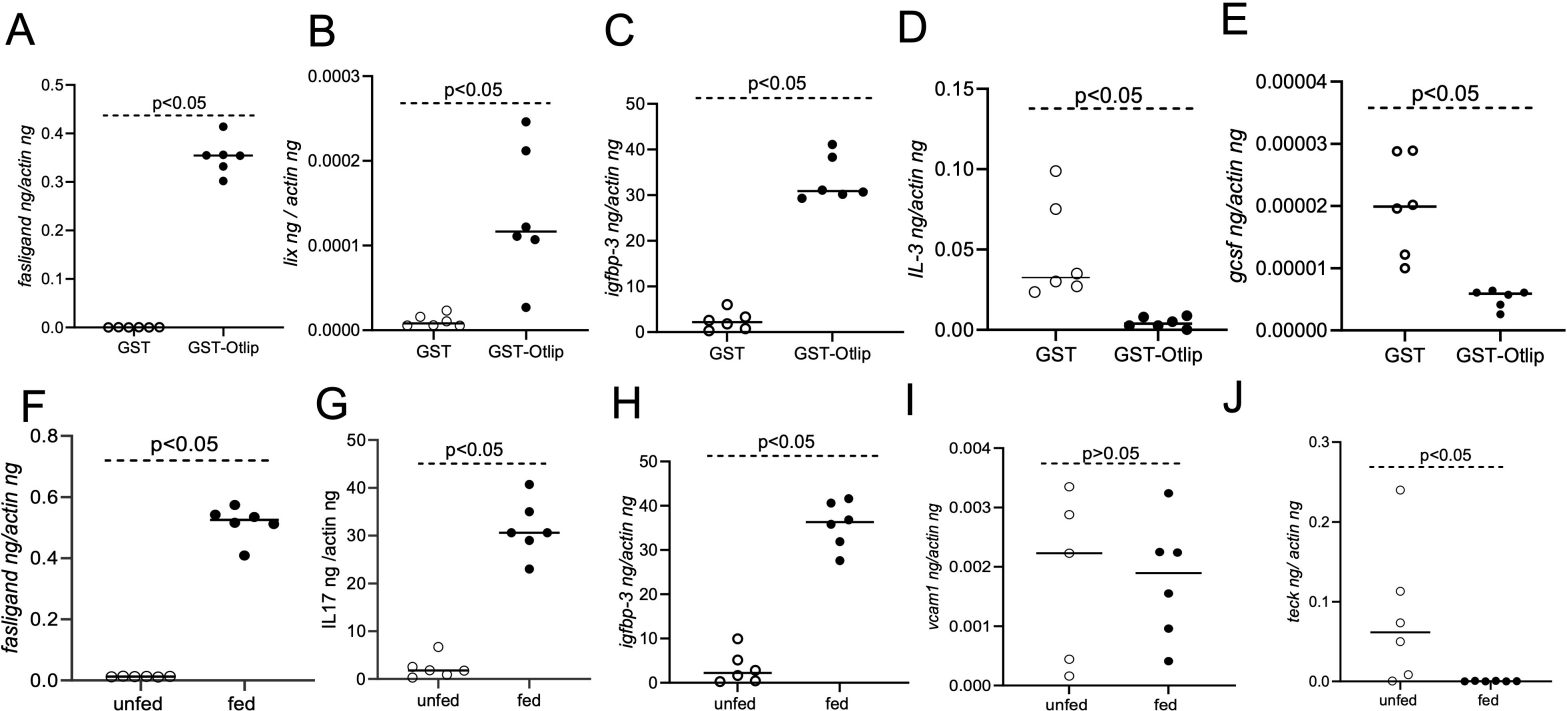
Treatment of murine macrophages with rGST-Otlip or salivary gland lysates modulates cytokine gene expression. qRT-PCR analysis showing transcript levels of *fas*-ligand **(A)**, *lix* **(B)**, *igfbp-3* **(C)**, *il*-3 **(D)** and *gcsf* **(E)** in murine macrophage cells upon treatment with rGST or rGST-Otlip. qRT-PCR analysis showing transcript levels of *fas*-ligand **(F)**, *il*-17 **(G)**, *igfbp-3* **(H)**, *vcam*-1 **(I)** and *teck* **(J)** in murine macrophage cells upon treatment with salivary gland lysates generated from unfed or fed *O. turicata americanus* ticks. Open circles indicate data from samples generated upon treatment with rGST or salivary gland lysates from unfed ticks and closed circles represent data from samples generated upon treatment with rGST-Otlip or salivary gland lysates from fed ticks. The transcript levels of cytokines were normalized to mouse actin transcript levels. Statistical analysis was performed using Mann-Whitney U test and P value is shown.

We further validated the protein array data by analyzing transcript levels upon treatment of murine macrophages with rGST/GST-Otlip or unfed/fed tick salivary gland lysates. RNA was extracted from these treated murine macrophages and processed for quantitative-real time PCR analysis (qRT-PCR analysis). Based on the cytokine protein array expression profile, we selected top three upregulated and bottom two downregulated genes in the rGST/rGST-Otlip or unfed/fed array tick salivary gland lysate treatment groups (**Figure 3**). The qRT-PCR analysis revealed significant (P<0.05) upregulation of *fas-ligand* (**Figure 3A**), *lix* (**Figure 3B**) and *igfbp3* (**Figure 3C**) transcripts in macrophages treated with rGST-Otlip compared to the levels noted upon treatment with rGST control. In addition, we noted significant (P<0.05) downregulation of *il-3* (**Figure 3D**) and *gcsf* (**Figure 3E**) transcripts upon treatment with rGST-Otlip compared to the levels noted upon treatment with rGST. Furthermore, treatment of murine macrophages with fed tick salivary gland lysates showed significant (P<0.05) upregulation of *fas-ligand* (**Figure 3F**), *il-17* (**Figure 3G**) and *igfbp3* (**Figure 3H**) transcripts compared to the levels noted upon treatment with unfed tick salivary gland lysates. No differences in the *vcam-1* (**Figure 3I**) transcript levels in murine macrophages were noted between unfed or fed tick salivary gland lysates treated groups. We also noted that treatment of murine macrophages with fed tick salivary gland lysates showed significant (P<0.05) downregulation in *teck* transcripts compared to the levels noted upon treatment with unfed tick salivary gland lysates (**Figure 3J**). These qRT-PCR results validated some of the proteins noted to be differentially secreted from macrophages upon treatment with rGST-Otlip or fed tick salivary gland lysates.

### Treatment with rGST-Otlip induces apoptotic gene expression in murine macrophages

**Figure 4:**
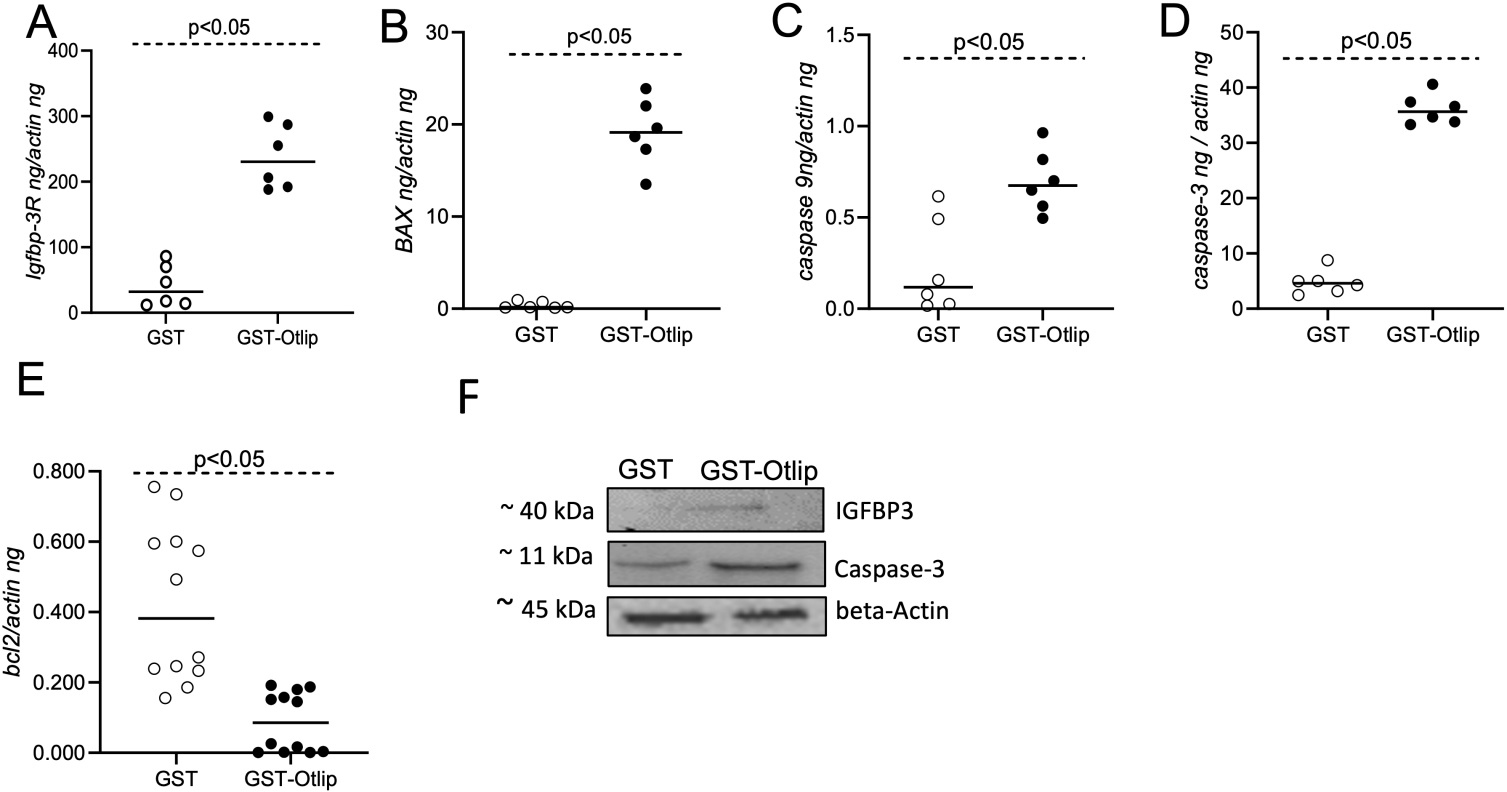
rGST-Otlip treatment causes significant upregulation of apoptotic gene expression in murine raw macrophages. qRT-PCR analysis showing expression of *igfbp-3R* **(A)**, *bax* **(B)**, *caspase-9* **(C)**, *caspase-3* **(D)**, and *bcl-*2 **(E)** in murine macrophage cells upon treatment with rGST or rGST-Otlip. The mRNA levels of cytokines were normalized to mouse actin mRNA levels. Statistical analysis was performed using Mann-Whitney U test and P value is shown. Open circles indicate data from samples generated upon treatment with rGST and closed circles represent data from samples generated upon treatment with rGST-Otlip. **(F)** Immunoblotting analysis showing levels of IGFBP3 and Caspase-3 in murine macrophage cells upon treatment with rGST or rGST-Otlip. Beta-actin levels serve as loading control in the immunoblotting analysis. Protein sizes are indicated as kilodaltons (kDa).

The observation of changed cell morphology (**Supplementary Figure 3**), secretion of increased levels of IGFBP-3 (**Figure 2**) and increased *igfbp3* transcripts in murine macrophages (**Figure 3C**) upon treatment with rGST-Otlip prompted us to further focus this study on this host molecule. IGFBP-3 binds to its receptor IGFBP-3R and is involved in inducing cell death (30–34). We therefore reasoned to test the effect of rGST-Otlip on *igfbp-3r* and apoptotic genes, and protein expression. qRT-PCR analysis showed significantly (P<0.05) higher transcript levels of *igfbp-3r* (**Figure 4A**) and the pro-apoptotic genes *bax* (**Figure 4B**), *caspase-9* (**Figure 4C**) and *caspase-3* (**Figure 4D**) upon treatment with rGST-Otlip compared to the transcript levels noted upon treatment with rGST control. In addition, we noted significantly (P<0.05) lower level of anti-apoptotic gene, *bcl-2*, upon treatment with rGST-Otlip compared to the levels noted upon treatment with rGST control (**Figure 4E**). Furthermore, immunoblotting analysis showed increased level of IGFBP-3 and Caspase-3 in macrophages upon treatment with rGST-Otlip compared to the levels noted upon rGST control treatment (**Figure 4F**; **Supplementary Figure 4**, **5**). These results show that rGST-Otlip induces pro-apoptotic genes and downregulates anti-apoptotic gene in murine macrophages.

### Treatment with fed *O. turicata americanus* salivary gland lysates induce apoptotic genes in murine macrophages

**Figure 5:**
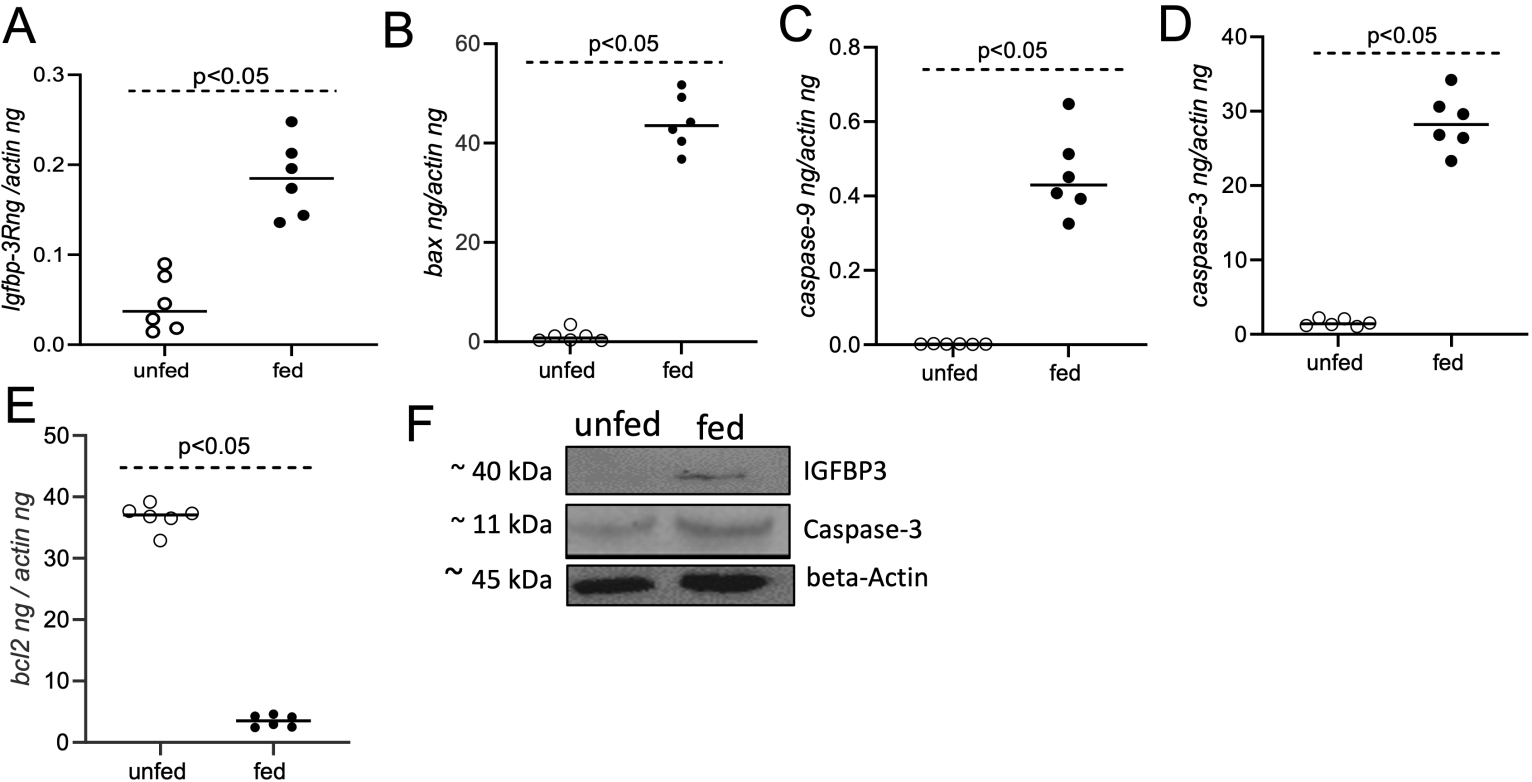
Treatment of murine macrophages with *O. turicata americanus* salivary gland lysate induces apoptotic gene expression. qRT-PCR analysis showing expression of *igfbp-3R* **(A)**, *bax* **(B)**, *caspase-9* **(C)**, *caspase-3* **(D)**, and *bcl-2* **(E)** transcripts in murine macrophages upon treatment with salivary gland lysates generated from *O. turicata americanus* unfed or fed nymphal ticks. The transcript levels of cytokines were normalized to mouse beta-actin transcript levels. Statistical analysis was performed using Mann-Whitney U test and P values are shown. Open circles indicate data from samples generated upon treatment with salivary gland lysates from unfed ticks and closed circles represent data from samples generated upon treatment with salivary gland lysates from fed ticks. **(F)** Immunoblotting analysis showing expression of IGFBP3 and Caspase-3 protein in murine macrophages upon treatment with *O. turicata americanus* unfed or fed salivary gland lysates. Beta-actin levels serve as loading control. Protein sizes are indicated as kilodaltons (kDa).

The expression levels of apoptotic genes were further quantified in murine macrophages upon treatment with salivary gland lysates generated from unfed or fed *O. turicata americanus* ticks. qRT-PCR analysis indicated significantly (P<0.05) higher expression levels of *igfbp-3r* (**Figure 5A**), and pro-apoptotic markers *bax* (**Figure 5B**), *caspase*-9 (**Figure 5C**) and *caspase-3* (**Figure 5D**) transcripts in murine macrophages upon treatment with salivary gland lysates generated from fed ticks compared to the levels noted upon treatment with salivary gland lysates generated from unfed ticks. In addition, we noted that transcript level of anti-apoptotic gene *bcl-2* (**Figure 5E**) were significantly (P<0.05) lower in murine macrophages upon treatment with salivary gland lysates generated from fed ticks compared to the levels noted upon treatment with salivary gland lysates generated from unfed ticks. Furthermore, immunoblotting analysis showed increased level of IGFBP-3 and Caspase-3 in macrophages upon treatment with salivary gland lysates generated from fed ticks compared to the levels noted upon treatment with salivary gland lysates generated from unfed ticks (**Figure 5F**; **Supplementary Figures 4**, **5**). Collectively, these results support the observations noted with rGST-Otlip in inducing pro-apoptotic genes and reducing anti-apoptotic gene expression in murine macrophages.

### Treatment with rGST-Otlip induces cell-death in murine macrophages

**Figure 6:**
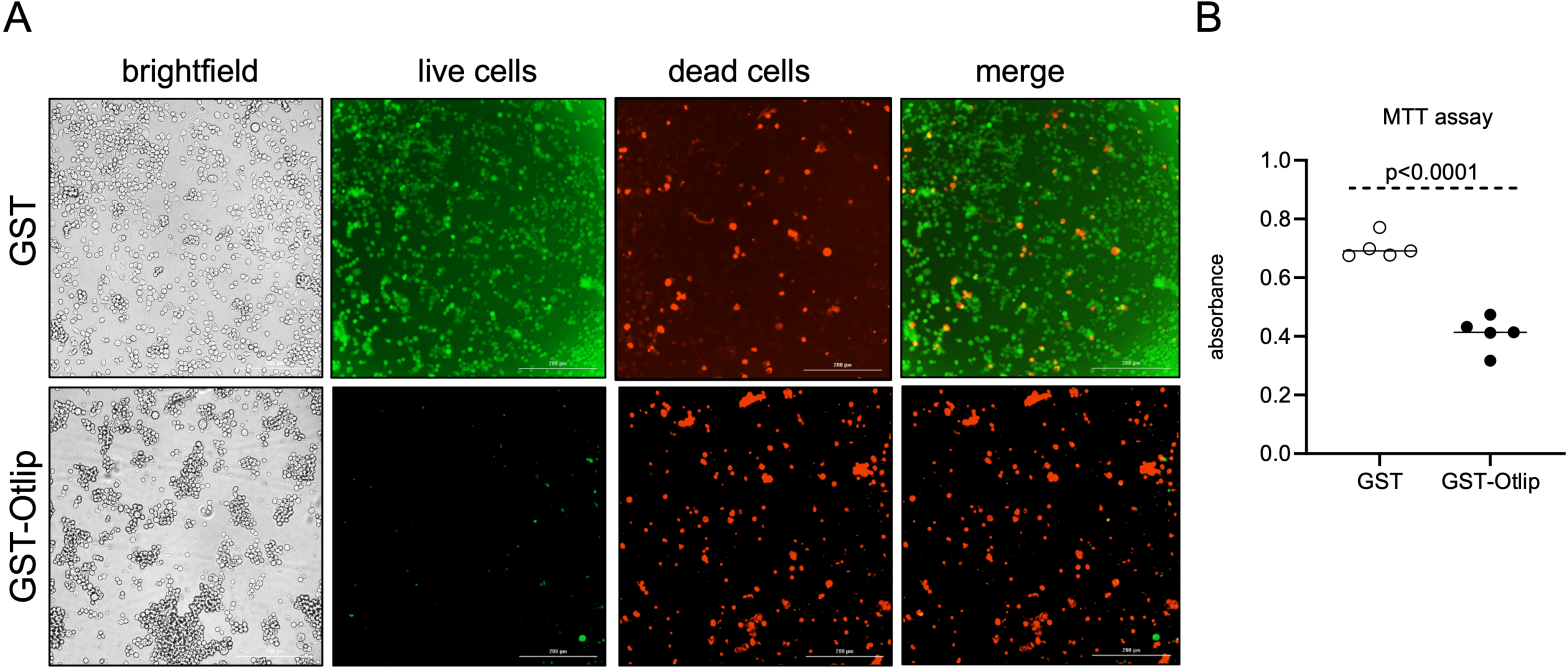
rGST-Otlip protein causes cell death in murine macrophages. **(A)** Representative Live/Dead-stained images of murine macrophages illustrating cellular viability and morphology at 24 hours post treatment with rGST and rGST-Otlip are shown. Brightfield images are shown for comparison. Green stained cells indicate live cells. Red stained cells indicate dead cells. Merge image indicates merge of live and dead cells. Images were captured using a fluorescence microscope (Cytation 7 imaging system) equipped with green and red fluorescence channels (Cytation 7 imaging system). Scale bar indicates 200 µm. Images were obtained at 20X magnification. **(B)** MTT assay results showing quantitative cell viability assessment upon treatment with rGST or rGST-Otlip at 5µg/mL concentration. Y-axis indicates absorbance obtained by subtracting absorbance at 690 nm from absorbance values at 570 nm. Data are presented as median of n=5 samples. Statistical analysis was performed using Mann-Whitney U test and P value is shown.

The observation of induced pro-apoptotic and reduced anti-apoptotic gene expression and protein levels in murine macrophages prompted us to further investigate whether treatment with rGST-Otlip protein has any effect on cell viability. We performed Live/Dead assay to determine the cell death upon treatment of murine macrophages with rGST-Otlip. Brightfield and fluorescent microscopy imaging showed a higher number of live murine macrophage cells upon treatment with control rGST (**Figure 6A**) compared to the number noted upon treatment with rGST-Otlip (**Figure 6A**). In contrast, we noted higher number of dead murine macrophage cells upon treatment with rGST-Otlip (**Figure 6A**) compared to the number noted upon treatment with rGST control (**Figure 6A**). Furthermore, the MTT assay corroborated the Live/Dead imaging data where the number of viable murine macrophage cells were significantly (P<0.05) reduced upon treatment with rGST-Otlip compared to number of viable cells noted upon treatment with rGST control (**Figure 6B**). These results show that rGST-Otlip induces cell death in murine macrophages.

### Treatment with rGST-Otlip induces IGFBP3 and apoptotic molecules in human keratinocytes

**Figure 7:**
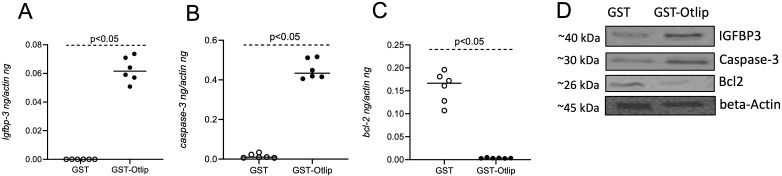
Otlip treatment modulates apoptotic gene and protein expression in human keratinocytes: qRT-PCR analysis showing expression of *igfbp-3* **(A)**, *caspase-3* **(B)** and *bcl-2* **(C)** in HaCaT cells upon treatment with rGST or rGST-Otlip. The mRNA levels of cytokines were normalized to human beta-actin mRNA levels. Open circles indicate data from samples generated upon treatment with rGST and closed circles represent data from samples generated upon treatment with rGST-Otlip. Statistical analysis was performed using Mann-Whitney U test and P value is shown. **(D)** Immunoblotting analysis showing expression of IGFBP3, Caspase -3 and BCL-2 proteins in HaCaT cells upon treatment with rGST or rGST-Otlip. Beta-actin levels serve as loading controls. Protein sizes are indicated as kilodaltons (kDa).

Since skin keratinocytes are a major part of the barrier between the tick-host interface and are among the first responders to tick bite (35), we wanted to investigate whether Otlip has any effects on apoptotic gene expression. We noted increased vacuolated cells upon treatment of human keratinocytic cell line, HaCaT cells, with rGST-Otlip compared to cells treated with rGST control (**Supplementary Figure 6**). qRT-PCR analysis showed significantly (P<0.05) higher expression of *igfbp3* (**Figure 7A**) and *caspase-3* (**Figure 7B**) transcripts in HaCaT cells upon treatment with rGST-Otlip compared to the levels noted upon treatment with rGST control. In addition, significantly (P<0.05) lower level of anti-apoptotic *bcl-2* gene expression was noted upon treatment of HaCaT cells with rGST-Otlip compared to levels noted upon treatment with rGST control (**Figure 7C**). Furthermore, immunoblotting analysis supported the qRT-PCR results (**Figure 7D**; **Supplementary Figures 7**, **8**). These results show that rGST-Otlip not only exert its effect on IGFBP3 and apoptotic signaling in macrophages but also in human keratinocytes.

### Treatment with rGST-Otlip induces cell-death in human keratinocytes

**Figure 8:**
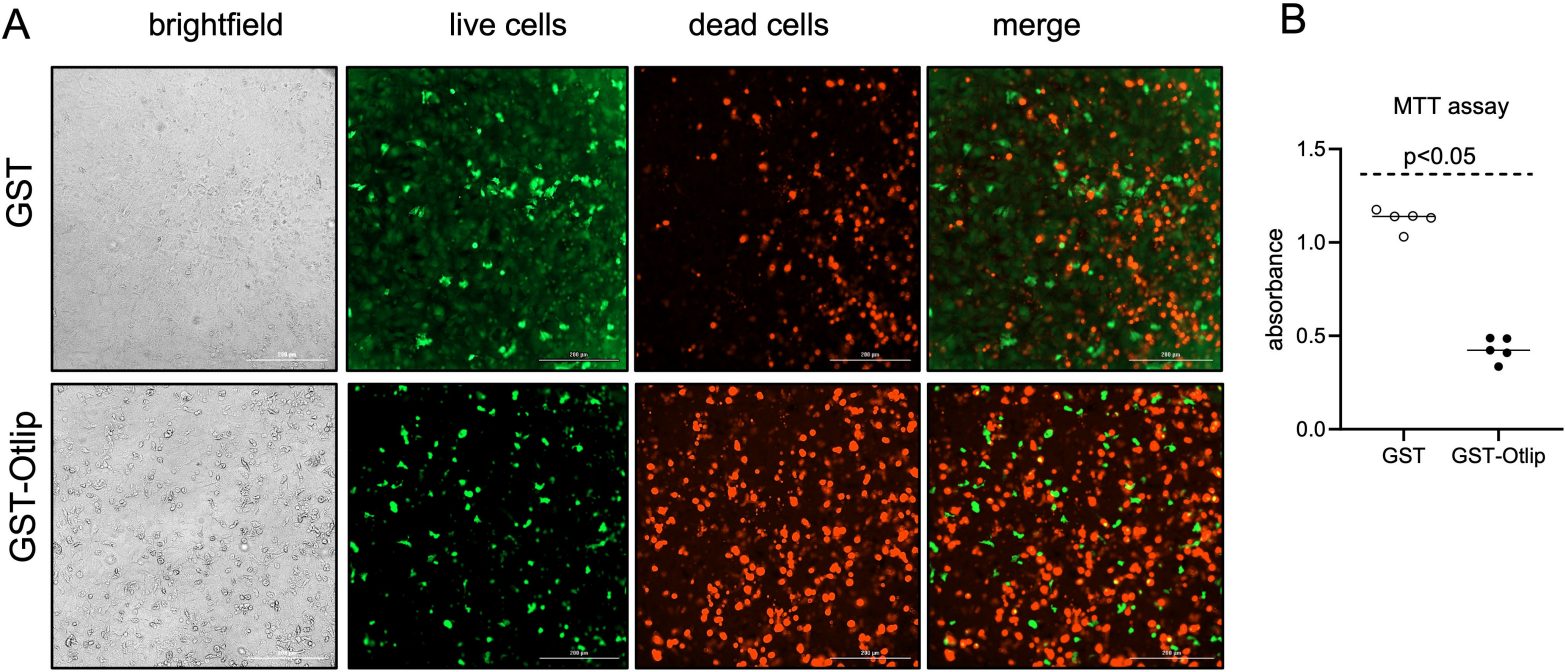
Treatment with rGST-Otlip protein causes cell death in human keratinocytes. **(A)** Representative Live/Dead-stained images of human HaCaT cells illustrating cellular viability and morphology at 24 hours post treatment with rGST and rGST-Otlip are shown. Brightfield images are shown for comparison. Green stained cells indicate live cells. Red stained cells indicate dead cells. Merged image indicates merge of live and dead cells. Images were captured using a fluorescence microscope (Cytation 7 imaging system) equipped with green and red fluorescence channels. Scale bar indicates 200 µm. Images were obtained at 20X magnification. **(B)** MTT assay results showing quantitative HaCaT cell viability assessment upon treatment with rGST or rGST-Otlip at 5µg/mL concentration. Y-axis indicates absorbance obtained by subtracting absorbance at 690 nm from absorbance values at 570 nm. Open circles indicate data from samples generated upon treatment with rGST and closed circles represent data from samples generated upon treatment with rGST-Otlip. Data are presented as median of n=5 samples. Statistical analysis was performed using Mann-Whitney U test and P value is shown.

We then performed Live/Dead assay to determine whether rGST-Otlip-mediated induction of apoptotic gene expression in HaCaT cells has any effect on cell viability. Microscopic analysis revealed increased number of viable HaCaT cells upon treatment with control rGST (**Figure 8A**) compared to the number of viable cells noted upon treatment with rGST-Otlip (**Figure 8A**). Like in murine macrophages, we noted increased number of dead HaCaT cells upon treatment with rGST-Otlip (**Figure 8A**) compared to the number of dead cells noted upon treatment with control rGST (**Figure 8A**). MTT assay further supported the Live/Dead imaging data where the number of viable HaCaT cells were significantly (P<0.05) reduced upon treatment with rGST-Otlip compared number of viable cells noted upon treatment with control rGST control (**Figure 8B**). Taken together, these results show that Otlip not only induces cell death in murine macrophages but also reveals similar effects in human keratinocytes.

### Treatment with rGST-Otlip induces apoptosis in murine macrophages

**Figure 9:**
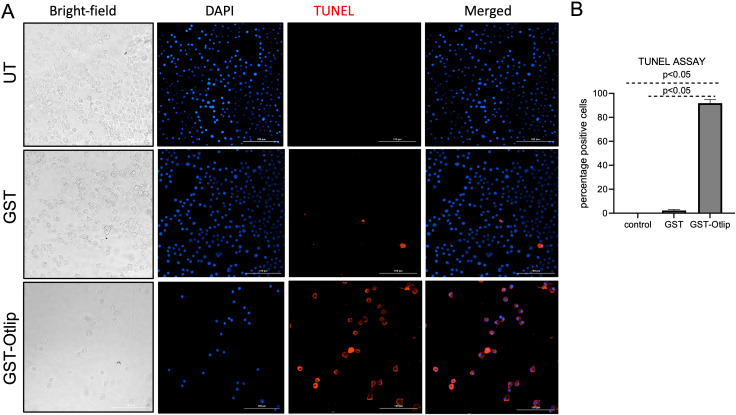
Treatment with rGST-Otlip causes apoptosis in murine macrophages. **(A)** Shown are the brightfield/DAPI/TUNEL/merged images of murine macrophages upon treatment with rGST or rGST-Otlip at 5µg/mL concentration. Untreated (UT) cells were used as another control. DAPI stained nuclei are indicated in blue color. TUNEL positive cells are indicated as red cells. Merged image shows merge of DAPI/TUNEL positive cells. Scale bar indicates 200 µm. Images were obtained at 20X magnification using Cytation 7 imaging system. **(B)** Quantification of percentage TUNEL positive cells upon treatment of murine macrophages with rGST or rGST-Otlip is shown. Y-axis indicates percentage of TUNEL positive cells per field. Data was generated based on readings obtained from 5 independent microscopic image fields. Statistical analysis was performed using Mann-Whitney U test and P value is shown.

For detection and visualization of the phenomenon of apoptosis elicited by tick Otlip protein in murine raw macrophages, we performed the TUNEL assay. Murine macrophages were treated with rGST control or rGST-Otlip and processed for TUNEL assay. Untreated macrophage cells were used as control. Based on the microscopic analysis, we noted higher numbers of TUNEL-positive macrophage cells upon treatment with control rGST-Otlip compared to the number noted upon treatment with rGST control or number noted in untreated cells (**Figure 9A**). Furthermore, we quantified the number of TUNEL-positive cells from several images and plotted data based on the percentage of TUNEL-positive cells from each group. The quantitative data shows significantly increased TUNEL-positive cells in murine macrophages upon treatment with rGST-Otlip compared to the number noted upon treatment with rGST control or number noted in untreated cells (**Figure 9B**). These data indicate that Otlip induces apoptosis in macrophages.

### siRNA-mediated silencing of *igfbp3* expression inhibits tick Otlip-mediated apoptosis in murine macrophages

**Figure 10:**
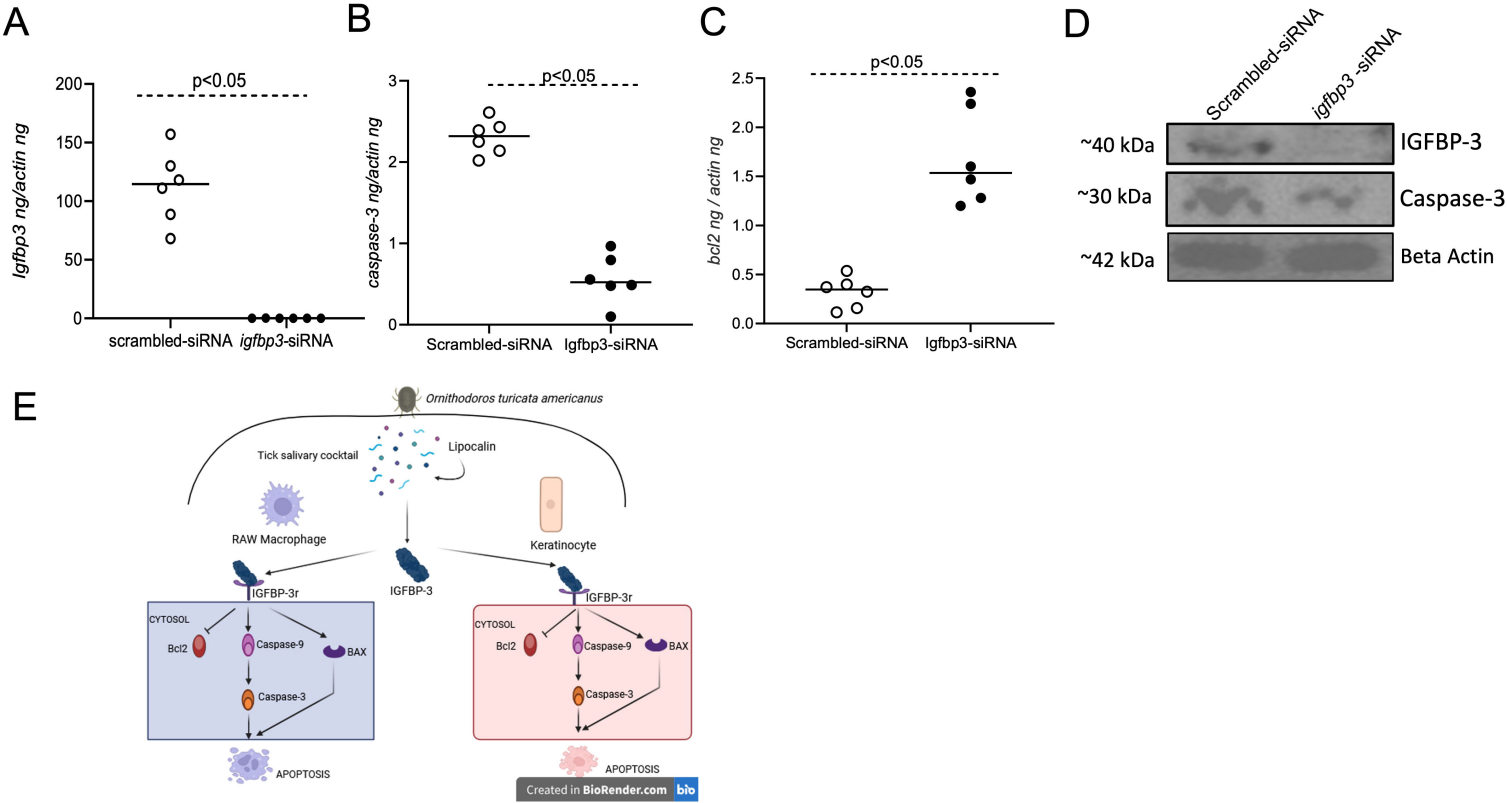
Knockdown of *igfbp3* expression affects rGST-Otlip-induced apoptosis in murine macrophages. qRT-PCR analysis showing expression of *igfbp3* **(A)**, *caspase-3* **(B)** and *bcl-2* **(C)** in *igfbp3/scrambled*-siRNA-rGST-Otlip-treated macrophages. The mRNA levels of *igfbp3* **(A)**, *caspase-3* **(B)** and *bcl-2* **(C)** were normalized to mouse beta-actin mRNA levels. Open circles indicate data from scrambled siRNA-treated group and closed circles represent data from *igfbp3*-siRNA-treated group. Statistical analysis was performed using Mann-Whitney U test and P values are shown. **(D)** Immunoblotting analysis showing expression of IGFBP3 and Caspase -3 proteins in *igfbp3/scrambled*-siRNA-rGST-Otlip-treated macrophages. Mouse beta-actin levels serve as loading controls. Protein sizes are indicated as kilodaltons (kDa). **(E)** Schematic representation showing the role of *O. turicata americanus* lipocalin in the modulation of apoptosis via IGFBP3 signaling in mammalian cells. While feeding on a mammalian host, ticks release salivary cocktail containing histamine-binding lipocalin. The secreted tick lipocalin stimulates the production of IGFBP-3 (Insulin-like growth factor-binding protein-3) and its receptor IGFBP-3R in both murine macrophages and human keratinocytes. The secreted IGFBP-3 could then bind to IGFBP-3R to stimulate apoptotic cascade. Otlip induces mammalian Caspase 9 (initiator caspase), Caspase 3 (executioner caspase) and repress BCL-2 (anti-apoptotic) to induce apoptosis in macrophages and keratinocytes. Induction in apoptosis of immune cells like macrophages and resident cells like keratinocytes would enable ticks to successfully complete their blood feeding.

We then performed siRNA-mediated silencing experiments to further analyze whether Otlip directly mediates IGFBP-3-associated signaling to induce apoptosis. Murine macrophages were first treated with *igfbp3*-siRNA or scrambled siRNA followed by treatment with rGST-Otlip. qRT-PCR analysis revealed significantly (P<0.05) reduced expression of *igfbp3* in *igfbp3*-siRNA-rGST-Otlip-treated murine macrophages compared to the levels noted in scrambled-siRNA-rGST-Otlip-treated cells (**Figure 10A**). In addition, we noted significantly (P<0.05) lower levels of *caspase-3* transcripts (**Figure 10B**) and higher levels of *bcl-2* transcripts in *igfbp3*-siRNA-rGST-Otlip-treated murine macrophages compared to the levels noted in scrambled-siRNA-rGST-Otlip-treated cells (**Figure 10C**). Furthermore, microscopic analysis showed increased cell death in scrambled-siRNA-rGST-Otlip-treated macrophages compared to *igfbp3*-siRNA-rGST-Otlip-treated cells (**Supplementary Figure 9**). Immunoblotting analysis supported qRT-PCR analysis (**Figure 10D**; **Supplementary Figure 10**). We noted lower levels of IGFBP3 and Caspase-3 proteins in *igfbp3*-siRNA-rGST-Otlip-treated murine macrophages compared to the levels noted in scrambled-siRNA-rGST-Otlip-treated cells (**Figure 10D**; **Supplementary Figure 10**). Taken together, these results indicate that *O. turicata americanus* tick lipocalin (Otlip) induces apoptosis in mammalian macrophages and keratinocytes by influencing IGFBP3-mediated signaling.

## Discussion

Arthropod lipocalins are noted to bind histamine and serotonins and reduce itching in the host during tick blood feeding (4, 8, 10, 15). So far, their role in mediating host cell death is not well understood. In this study, we provide evidence for the role of tick lipocalins in modulating cytokines and inducing mammalian IGFBP3-mediated apoptosis in murine macrophages and human keratinocytes.

We selected murine macrophage cell line in this study because these cells are important in forming an innate immune barrier in the skin (36). Macrophages are usually present in the superficial region of dermis (36). Skin macrophages help to defend against invading pathogens that enter through the sites of damage in the skin (36). These cells are involved in phagocytosis, production of cytokines, chemotaxis and could serve as antigen presenting cells (36). In addition, *Dermacentor variabilis* tick salivary components are shown to modulate the migration and cytokine profile in these cells (37). We also included the human keratinocyte cell line in this study mainly because these are the first line of cells that contact the tick mouth parts during arthropod blood feeding (35). Therefore, it is likely that tick salivary proteins may have direct contact with these cells. Keratinocytes are the principal epidermal cells that are reported to produce many pro- and anti-apoptotic molecules (38). Recently, using the same human keratinocyte cell line that was used in this study, we reported that exosomes derived from tick saliva and salivary glands inhibit/delays wound healing and repair process (11). We also reported that tick saliva/salivary gland derived exosomes modulate CXCL12 and IL-8 chemokines (11). In addition, several studies have reported that tick saliva modulates cytokine responses from keratinocytes (39–41). The observation of increased IGFBP3 levels, induction of pro-apoptotic markers and reduced levels of an anti-apoptotic molecule indicates that Otlip could induce apoptosis in both macrophages and keratinocytes. We performed several experiments with cells plated in different plates and in different volume of culture media. The plate size and volume of the media was chosen based on the experimental need. However, we maintained rGST and rGST-Otlip concentration at the same level for treatments in different experimental conditions. Our future studies will focus to test the effect of different concentrations of these proteins in different experimental conditions. The data obtained from all different experiments in this study suggested Otlip-mediated and IGFBP3-associated effects in these cells. Furthermore, the data from siRNA-mediated silencing experiments further supports that Otlip induces apoptosis in macrophages and keratinocytes by influencing the IGFBP3-associated signaling. We believe that apoptosis of immune and/or resident cells, such as keratinocytes, at the bite site could disrupt the host’s immune defenses and aid in successful tick blood feeding.

IGFBPs family are evolutionarily conserved, cysteine-rich extracellular secretory proteins found across a broad range of organisms, including humans, fish, chickens, fruit flies, army worms, and shrimps (42). IGFBPs are integral part of the insulin-like growth factor (IGF) signaling system (43). While IGFBPs are traditionally known for their role in binding and modulating IGF function and related signaling, recent research has uncovered that some IGFBPs, particularly IGFBP-3, may exert significant IGF-independent effects (33). There are six known types of IGFBPs reported (IGFBP-1 to 6), of which IGFBP-3 is the most abundant in the bloodstream (44). The observation of increased secretion of IGFBP-3 from both murine macrophages and human keratinocytes upon treatment with Otlip suggests that this tick protein impacts the secretion of the most abundant IGFBP member. We selected IGFBP-3 for the detailed analysis because of two reasons. First, this protein was noted to be one among the top five that are highly secreted host proteins upon treatment of murine macrophages with rGST-Otlip or fed tick salivary gland lysates. Second, the observation of increased cell death upon treatment with rGST-Otlip (**Figure 8**) and a study reporting involvement of this protein in the inhibition of cell proliferation and modulation of apoptosis (45). Additionally, FAS-ligand was noted to be the top highly secreted protein from murine macrophages upon treatment with rGST-Otlip or fed tick salivary gland lysate. FAS-ligand binds to FAS-receptor and mediates signaling that induces apoptosis in mammalian cells (46). Therefore, observation of increased secretion of both IGFBP3 and FAS-ligand from murine macrophages strongly support the overall conclusion of this work that tick Otlip induces apoptotic signaling in mammalian cells.

Apoptosis, or programmed cell death, is an important phenomenon for the normal development, homeostasis, and disease states (47, 48). Recent studies have identified IGFBP-3 receptor (IGFBP-3R), a new cell death receptor that is specifically bound by IGFBP-3 (42, 45, 49). We noted increased transcripts of IGFBP-3R in murine macrophages upon treatment with rGST-Otlip. IGFBP-3R interacts directly with caspase-8, facilitating its activation (42, 45, 49). This interaction was suggested to be activated upon IGFBP-3 binding, leading to subsequent activation of executioner caspases, including caspase-3 (42, 45, 49). Our qRT-PCR and immunoblotting results showed significantly higher levels of caspase-3 transcripts and protein, respectively, suggesting that Otlip could induce IGFBP3-mediated apoptosis in a similar mechanism.

A study has shown that *Amblyomma americanum* ticks and other hard ticks also encode IGFBP-related proteins (50). These proteins are highly conserved among tick species and show high degree of identity in their amino acid sequence (50). The signature motif CGCCXXC of amino terminus region of mammalian IGFBPs are conserved in ticks (50). RNAi-mediated silencing of tick IGFBP-related proteins affected tick feeding (50). The role of tick IGFBP-related proteins in inducing apoptosis in mammalian cells is not known. However, the observation of reduced tick feeding upon silencing of tick IGFBP suggests that these proteins may also be required in inducing apoptosis of immune or resident cells at the tick bite site. Future studies could unravel some of these interesting observations on tick IGFBP-related proteins.

The BCL-2 family of proteins are classified in to three sub-categories: BCL-2 and BCL-XL group that have anti-apoptotic function, BAX and BAK that have pro-apoptotic function and BAD and BID proteins that have pro-apoptotic function (51). The data from qRT-PCR analysis showing increased *bax* transcript levels and reduced *bcl-2* transcript levels in murine macrophages upon rGST-Otlip treatment further support their roles as pro-apoptotic and anti-apoptotic genes, respectively. Based on all the findings, we propose a model for Otlip-stimulated IGFBP3-mediated apoptosis in macrophages and keratinocytes. During blood feeding *O. turicata americanus* secrete Otlip in saliva (**Figure 10**). Otlip stimulates production of IGFBP-3 from macrophages and keratinocytes by an unknown mechanism. The increased production of IGFBP-3 could lead to increased binding to IGFBP-3R thereby stimulating downstream activation of Caspase-9 and Caspase-3 (**Figure 10**). In addition, binding of IGFBP-3 to IGFBP-3R could also downregulate anti-apoptotic BCL-2 and upregulate proapoptotic BAX (**Figure 10**) proteins. All these processes could lead to induction of apoptosis in macrophages and keratinocytes.

In summary, our study provides evidence for the role of tick lipocalins in modulating cytokine/chemokine response from macrophages and keratinocytes. The findings from this study not only underscores a novel mechanism for a tick lipocalin in inducing IGFBP-3-mediated apoptosis of mammalian cells but also could lead to the development of anti-tick-lipocalin-based strategies to prevent tick feeding.

## Materials and methods

### Ticks, mice and salivary gland and gut dissections

Laboratory-reared uninfected *Ornithodoros turicata americanus* ticks were used in this study. These ticks were provided by Dr. J.H. Oliver, Jr. from Georgia Southern University, Statesboro, GA, to Dr. Daniel Sonenshine at Old Dominion University (ODU), VA. The colony was maintained at ODU. A detailed description of these specimens is provided in our previous publications (52, 53). To generate fed nymphs, unfed nymphs were fed on naïve 6–8 weeks-old CD1 mice (Charles River Laboratories, USA). Fed repleted ticks were collected and processed for dissections of salivary glands. In addition, salivary gland tissues were also isolated from unfed nymphs. These tissues were dissected in 1X PBS and total protein lysates were prepared in complete RIPA lysis buffer (ThermoFisher Scienctific, USA). Protein concentration was measured using BCA protein assay kit (ThermoFisher Scientific, USA).

### Ethics statement

Tick feeding studies on animals was performed based on the approved Institutional animal care and use committee (IACUC) protocol (10–018) at Old Dominion University. During tick feeding, Acepromazine was used as a tranquilizer to minimize discomfort/anxiety in animals.

### Tick Otlip and GST protein purification

We previously identified and cloned the lipocalin-like molecule, *otlip*, from the soft ticks *Ornithodoros turicata americanus* (22). *Escherichia coli* clones were inoculated in 3 mL Luria Broth (LB) containing 50 microgram/mL of ampicillin and incubated overnight in a shaker. The overnight culture was used to inoculate a fresh 50 mL LB with ampicillin, and the cells were allowed to grow till optical density (OD)_600_ between 0.5-0.6. The protein expression was induced by addition of 1mM Isopropyl β-D-1-thiogalactopyranoside (IPTG) and *E. coli* cells were allowed to grow at 37°C in a shaker for up to an hour or two for the OD_600_ to reach 1-1.2. After this, the cells were centrifuged at 4200 rpm for 15 minutes. To these pelleted bacterial cells, 1 mL Lysis buffer containing 1X PBS, 0.2 mg/mL lysozyme, 10 mM beta mercaptoethanol, 1% Triton X-100 and with 1mM PMSF was added and incubated in a shaker at 37°C for one hour. In the next step, sonication was performed (for ~20 secs) with intervals of ice incubation and pulsing. After 5–6 repeated round of sonication, the culture was centrifuged at 15000 rpm for 15 mins at 4°C. The supernatant was collected and to this 0.5–1 mL glutathione resin (GBiosciences, USA) was added and kept overnight at 4°C in a rotor/shaker. Next day, the mixture was pelleted by centrifugation at 2000g for 5 min. The pellet was mixed with 1 mL of wash buffer and transferred to a 5 mL disposable polypropylene column (ThermoScientific, USA) and placed in a 15 mL tube. Following 3 washes (by centrifugation of the assembly at 2000g for 3 minutes), 1 ml elution buffer was added and incubated at room temperature for 5 minutes and centrifuged at 2000g for 3 minutes. This was repeated 3 times to get 3 ml of rGST or rGST-Otlip. In the next step, dialysis was performed overnight with 1X PBS using Slide-A-Lyzer dialysis cassettes (ThermoScientific, USA). The dialyzed solutions (containing rGST or rGST-Otlip) were filtered through 10-KDa cut-off filter for obtaining concentrated and purified recombinant rGST (~26 kDa) and rGST-Otlip (~32 kDa). Protein concentration of dialyzed recombinant proteins was measured using Pierce BCA protein assay kit (ThermoFisher Scientific, USA). The proteins were loaded onto 12% SDS-PAGE gel to check quality of the purified proteins (**Supplementary Figure 1**).

### Cell culture and treatment

RAW 264.7 macrophage (ATCC, USA) and HaCaT cells (Fisher Scientific, USA) were seeded at a density of 1e5 cells per well in six replicates for each treatment in 12-well plates. Next day, these cells were treated with either 5 μg/mL/well of salivary gland lysates from unfed or fed ticks, or with rGST or rGST-Otlip. After 24 hours post treatment, cells were visualized under the microscope. In addition, cells were collected for RNA and protein extractions in RNA lysis buffer (BioRad, USA) or RIPA lysis buffer with protease inhibitor (ThermoFisher Scientific, USA), respectively. Cell culture supernatants were also collected and processed for Cytokine Array analysis.

### Cytokine arrays

Cytokine array analysis was performed using RayBiotech C-series Mouse Cytokine Antibody Array C3 (RayBiotech, USA) and with supernatants collected from RAW macrophages treated with salivary gland lysates from unfed or fed ticks or with rGST rGST-Otlip proteins. The Mouse Cytokine Array C3 (Ray Biotech Inc.) consisted of 62 different cytokine and chemokine antibodies spotted in duplicate onto a membrane. Four individual membranes were used in this study with one membrane/treatment group. All incubations and washes were performed under gentle rocking conditions at room temperature following manufacturer instructions. Care was taken to avoid appearance of bubbles on or between the membranes to ensure even distribution of the samples. Firstly, the membranes were blocked with 2 mL of blocking buffer and incubated at room temperature. Following aspiration of the buffer, 1 mL of supernatant (pooled from 6 replicates) was added to each array membrane and incubated overnight at 4°C. Next day, the samples were aspirated and 2 mL of 1X Wash Buffer I was added to each well and incubated for 5 minutes at room-temperature. This was repeated 2 more times for a total of 3 washes using fresh buffer and aspirating out the buffer completely each time. Similar wash steps were performed with 2 mL of 1X Wash Buffer II. In the next step, overnight incubation with biotinylated antibody cocktail was done under gentle rocking condition. Following this step, on the next day, similar wash steps with Wash Buffer I and II were repeated and then 2 mL of 1X HRP-Streptavidin was added to each well and incubated overnight at 4°C under gentle rocking condition. Next day, the solution was aspirated followed by washes with Wash Buffer I and II. The two individual membranes were placed side by side in a plastic protective folder and 500 µl of Detection Buffer (250 µl Detection Buffer C + 250 µl Detection Buffer D) was gently pipetted onto each membrane and incubated at room temperature for 2 minutes. Another plastic sheet was placed on top of the membranes by gently rolling the flexible plastic sheets such that the two membranes become sandwiched between the two plastic folders. The sandwiched array membranes were then imaged using chemiluminescence imaging system (BioRad, USA). Exposure times ranged from 30–60 seconds. The relative intensity densities indicate the change in the expression levels of the cytokines/chemokines. The intensities of the spots were quantified using Image-J software. Following subtraction of the negative control and normalization to the positive controls, integrated density value (IDV) for each cytokine in rGST-Otlip-treated or fed tick salivary gland lysate-treated samples was compared to the corresponding spot in the controls (rGST- or unfed-tick salivary gland lysate-treated samples). Fold-change values for all molecules in the cytokine array were calculated using IDVs and imported into Power BI (Microsoft, Redmond, WA, USA). Molecules were sorted in descending order based on their respective fold-change values, and a histography was generated with fold-change values used for the length and label for each bar. A color gradient was applied to the bars, with the neutral value (fold change = 1) shown in yellow, the most upregulated molecule in red, and the most downregulated molecule in blue.

### RNA extractions and quantitative real-time PCR analysis

Total RNA from RAW macrophage and HaCaT cell samples were generated using the Aurum Total RNA mini kit (Bio-Rad, USA) following the manufacturer’s instructions. RNA (200–250 ng) was converted to cDNA in a 10 µl reaction using BioRAD cDNA synthesis kit (BioRAD, USA) or QSCRIPT cDNA kit (QuantaBio/VWR, USA). One microliter of the generated cDNA was used as a template in each qRT-PCR reaction for quantifying transcript levels of cytokines/chemokines. Oligonucleotides used for quantifying mouse beta actin, IGFBP-3, IGFBP-3R, LIX, VCAM-1, TECK, IL-17, FAS ligand, G-CSF and IL-3 are published previously (54–63). Oligonucleotides used for quantifying human beta actin, IGFBP3, BCL-2 and Caspase-3 are previously published (11, 64–67). All other oligonucleotides used in this study are mentioned in **Supplementary Table 1**. The levels of all transcripts were normalized to levels of mouse or human actin transcript levels. QRT-PCR was performed using CFX96-Opus QPCR machine (BioRad, USA) and iQ-SYBR Green Supermix (BioRad, USA) or MAXIMA SYBR/R QPCR mix (Fisher Scientific, USA) as described (53, 68). In qRT-PCR reactions, the standard curve was generated using 10-fold serial dilutions of known quantities of respective fragments. Total RNA from RAW macrophage and HaCaT cells was used to generate cDNA which was the template for initial amplification of the genes. The qRT-PCR products were then run on 1.2% agarose gel, and the fragments were then excised, concentration was measured and sent for sequencing at Eurofins Genomics (USA). Standards were made with the purified fragments for each gene and diluted starting from 1 ng to 0.00001 ng.

### Immunoblotting analysis

Macrophages and HaCaT cells treated with either salivary gland lysates generated from unfed or fed ticks or with rGST or rGST-Otlip were collected and lysed in RIPA lysis buffer with protease inhibitor. Immunoblotting analysis was performed as described (68, 69). Total protein lysates (25 µg) from these cells were separated on 12% SDS-PAGE gels for IGFBP3, Bcl2 and beta-Actin (control) and 15% SDS-PAGE gel for Caspase-3 immunoblotting analysis. After gel electrophoresis, blots were blocked with 5% milk buffer and probed with IGFBP3 rabbit polyclonal antibody (cat. no. A16052, ABclonal, USA) or Caspase-3 p12 Rabbit monoclonal antibody (cat. no. A19664, ABclonal, USA) or Bcl2 Rabbit polyclonal antibody (cat. vo. A0208, ABclonal, USA) or beta-Actin rabbit monoclonal antibody (cat. no. 8457S, Cell Signaling Technologies, USA), followed by anti-rabbit HRP-conjugated secondary antibodies (cat. no. 7074S, Cell Signaling Technologies, USA). Antibody binding was detected with in-house ECL reagent and hydrogen peroxide (H_2_O_2_). ECL reagent was made in the lab based on the information given in the following link, https://bitesizebio.com/8970/how-to-make-your-own-ecl/ and the protocol was modified to make it user-friendly and to increase the shelf life of the prepared reagents. Briefly, 1 mL of 250 mM luminol (Sigma, USA) dissolved in DMSO and 0.44 mL of 90 mM p-Coumaric acid (Sigma, USA) dissolved in DMSO were mixed with 10 mL of 1 M Tris-Cl, pH:8.5. The ECL mixture was stored at -20 °C as 1 mL aliquots. This 1 mL aliquot was mixed with 7.5 mL deionized water and stored at -20 °C as 1 mL aliquots of ECL reagent A. Then 1 mL of ECL reagent A was mixed with ECL reagent B (4 µl of 3% H_2_O_2_) for chemiluminescence detection. Blots were imaged using Bio-Rad Chemidoc imaging system and processed using Image Lab software.

### Brightfield microscopy and live/dead assay:

After 24-hours post-treatment, cells were visualized under the Cytation 7 imager for the detection of any changes in cellular morphology. Images were taken on several distinct focal planes for each treatment group.

For Live/Dead assay, RAW macrophage and HaCaT cells were plated in a 96 well plate as five replicates for each treatment. Cells were plated at 1e4 cells per well. The cells were treated with 5 μg/mL/well rGST or rGST-Otlip. After 24 hrs post-treatment, the Live/Dead assay was performed using LIVE/DEAD™ Cell Imaging kit, Invitrogen as described in our previous study (69). Briefly, 1 mL of green dye was added to and mixed with the red dye and 25 μl was added to each well. The wells were incubated for 30 mins at 37°C. The wells were then visualized with Cytation 7 imager (BioTek, USA). The images of cells from green channel (488 nm) indicated the live/viable cells, and the cells in red channel (594 nm) indicated the dead cells.

### MTT assay

MTT assay was carried out as five replicates in a 96-well plate as described (69). The cells were seeded at a density of 3e4 per well containing 200 µl medium. After 24 hours of treatments with 5 μg/mL/well rGST or rGST-Otlip, 20 μl aliquots of MTT solution (5 mg/mL in 1x PBS) were added to each well. After three hours of incubation at 37°C and 5% CO2, the culture medium with MTT solution was removed, and formazan crystals were solubilized with 100 μl DMSO. The plates were then read on a microplate reader in CYTATION7 imaging system (BioTek, USA) at 570 nm and 690 nm wavelengths. Graphs were plotted by subtracting the absorbance value at 690 nm from the absorbance value at 570 nm.

### siRNA silencing

The siRNA for silencing *igfbp3* expression and scrambled siRNA (control) were obtained from Santa Cruz Biotechnology (USA). The catalog numbers for *igfbp3* and control siRNAs are sc-39588 and sc-37007, respectively. The gene specific siRNA products from Santa Cruz Biotechnology (USA) usually have pools of three to five target-specific 19–25 nt siRNAs. As per the company (Santa Cruz Biotechnology, USA) note, the control siRNA consists of a scrambled sequence that will not lead to the degradation of any specific cellular transcript. Murine raw macrophage cells were seeded to a density of 1e5 cells per well in a 12 well plate in 10% FBS supplemented RPMI media. Cells were incubated overnight at 37°C. Transfections were carried out as per the manufacturer’s instructions (Santa Cruz Biotechnology siRNA transfection protocol). Briefly, for each transfection, 2 µl of solution A (siRNA duplex) was diluted in 100 µl siRNA transfection medium. Similarly, 6 µl (peak activity as per the company’s recommendation) of solution B (transfection reagent) was diluted in 100 µl transfection medium. In the next step, the siRNA duplex solution or solution A was pipetted directly to the diluted solution B and the mixture was incubated for 30 minutes at room temperature. This was done for both the scrambled-siRNA and *igfbp3-*siRNA. The cells were washed with 1 mL of 1X PBS. Six wells were transfected with scrambled siRNA, and another six wells were transfected with *igfbp3* siRNA. For each transfection, 500 µl of solution A+B mixture was overlayed onto the washed cells and the cells were incubated with siRNA for 6 hours. After 6 hours, the cells were analyzed and we then added 1 mL of RPMI with 2X FBS without removal of the transfection mixture and the cells were incubated overnight at 37°C. Next day, the medium containing the siRNA was aspirated from each well and further washed with 1 mL of 1X PBS. Fresh culture medium was added to the wells and subsequently 5µg/mL/well purified rGST-Otlip was added and incubated overnight at 37°C. The following day, imaging was performed in Cytation-7 imaging system. The samples (six replicates) were collected in RNA lysis buffer for RNA extractions or RIPA buffer containing protease inhibitor for protein extractions.

### TUNEL assay

The TUNEL assay was performed using Click-iT Plus TUNEL assay kit (ThermoFisher Scientific, USA) following the manufacturer’s instruction. Briefly, cells were plated at a confluence of 1e5 cells per well in a 12 well plate. Next day, these cells were treated with 5 µg/mL/well rGST or rGST-Otlip. The TUNEL assay was performed 24 hours post treatment. In the first step, cells were fixed with 4% PFA (paraformaldehyde) and incubated at room temperature for 15 minutes. In the next step, cells were permeabilized with 0.25% Triton-X-100 in 1X PBS and incubated at room temperature for 20 minutes. Following two washes with 1X PBS the TdT reaction was performed by addition of 100 μl TdT reaction buffer per well and incubated for 10 minutes at 37°C. After a subsequent PBS wash, the Click-iT Plus TUNEL reaction cocktail was added to each well and incubated at 37 degrees Celsius for 30 minutes. In the final step, the reaction cocktail was removed, and washes were performed with 1X PBS solution. Following the washing step, imaging was performed immediately using Cytation 7 imager (BioTek, USA). The Alexa Flour 594 picolyl azide dye was the fluorophore used for detection and imaging of apoptosis.

### Statistical analysis

Statistical analysis was performed using GraphPad Prism 9 software. To compare two groups, Mann-Whitney U test was used. In the analysis, P<0.05 was considered significant. Horizontal lines in the scatter plots represent the median value. Wherever necessary, statistical test and P values used are reported.

## Data Availability

The original contributions presented in the study are included in the article/**Supplementary Material**. Further inquiries can be directed to the corresponding author.
