# Supplementary material for: Tick lipocalin triggers mammalian IGFBP-3-mediated apoptosis in macrophages and keratinocytes
Source: Front Immunol. 2026 Feb 18;17:1768484. doi: 10.3389/fimmu.2026.1768484 (PMC12956797; doi:10.3389/fimmu.2026.1768484)
Supplement: Supplementary file 1 [file Image1.pdf]

Tick lipocalin triggers mammalian IGFBP-3-mediated apoptosis in macrophages and keratinocytes

Krittika Nandy [1,2], P P Mahesh [1], Lichao Liu [1,3], Daniel E. Sonenshine [4, 5], Hameeda Sultana [1], and Girish Neelakanta [1, $]

[1] Department of Biomedical and Diagnostic Sciences, College of Veterinary Medicine, University of Tennessee, Knoxville, TN, USA. [2] Present address: Department of Biology, School of Health, Life Sciences and Education, Ohio Northern University, Ada, OH, USA. [3] Present address: Department of Ophthalmology, Indiana University School of Medicine, Indianapolis, IN, USA. [4] Department of Biological Sciences, Old Dominion University, Norfolk, VA, USA. [5] Vector Molecular Biology Section, Laboratory of Malaria and Vector Research, National Institute of Allergy and Infectious Diseases, National Institutes of Health, Rockville, MD, USA.

Supplementary information

Supplementary figure legends

Supplementary Figure 1: Purification of recombinant GST and GST-Otlip proteins. Coomassie stained gel images showing purified rGST or rGST-Otlip from *Escherichia coli* BL21 cell lysates. M indicates marker. Protein sizes are shown in kilodalton.

Supplementary Figure 2: RayBiotech C-series mouse cytokine array, C3 (Catalog # AAM-CYT-3-2) layout. Table shows the order of the cytokines and chemokines spotted on the nitrocellulose membrane utilized in the array. The capture antibodies are vertically spotted in duplicates onto the nitrocellulose membrane to allow high-throughput simultaneous multi-analyte profiling of 62 mouse cytokines and chemokines.

Supplementary Figure 3: Brightfield images of RAW macrophage pre- and post-treatment with rGST or rGST-Otlip or unfed/fed tick salivary gland lysates. Brightfield microscopic images showing murine macrophages before (pre-treatment) or after (post-treatment) with rGST or rGST-Otlip (A) or treatment with unfed or fed salivary gland lysates (B) treatment. Scale bar indicates 200 µm.

Supplementary Figure 4: Full-length immunoblot images for apoptotic markers in murine macrophages. Full-length immunoblot images showing detection of IGFBP-3 (A) and Caspase-3 (B) in murine macrophages treated with unfed or fed tick salivary gland lysates or treated with rGST or rGST-Otlip. Black arrows denote the protein of interest. The cropped sections of these images are shown in the main Figures 4 and 5.

Supplementary Figure 5: Full-length immunoblot images for beta-actin levels in murine macrophages. Full-length immunoblot images showing detection of beta-actin in murine macrophages treated with unfed or fed tick salivary gland lysates (A) or treated with rGST or rGST-Otlip (B). Black arrows denote the proteins of interest. The cropped sections of these images are shown in main Figures 4 and 5.

Supplementary Figure 6: Brightfield images of human keratinocyte cell line (HaCaT cells) pre- and post-treatment with rGST or rGST-Otlip. Brightfield microscopic images showing HaCaT cells before (Pre-treatment) or after (post-treatment) rGST or rGST-Otlip (A) treatment. Scale bar indicates 200 µm.

Supplementary Figure 7: Full-length immunoblot images for apoptotic markers in HaCaT cells. Full-length immunoblot images showing detection of IGFBP-3 (A), Caspase-3 (B) and BCL-2 (C) in HaCaT cells treated with rGST or rGST-Otlip. Black arrows denote the protein of interest. The cropped sections of these images are shown in main Figure 7.

Supplementary Figure 8: Full-length immunoblot images for beta-actin levels in HaCaT cells. Full-length immunoblot images showing detection of beta-actin in HaCaT cells treated with rGST or rGST-Otlip. Black arrows denote the protein of interest. The cropped sections of these images are shown in main Figure 7.

Supplementary Figure 9: Brightfield images of murine macrophages pre- and post-treatment with *igfbp3/scrambled*-siRNA and rGST-Otlip. Brightfield microscopic images showing murine macrophage cells before (pre-siRNA) or after (post-siRNA) and post rGST-Otlip treatment is shown. Scale bar indicates 200 μm.

Supplementary Figure 10: Full-length immunoblot images for apoptotic markers and beta-actin levels in macrophage cells after *igfbp3/scrambled*-siRNA and rGST-Otlip treatment. Full-length immunoblot images showing detection of IGFBP3 (A) and Caspase-3 (B) and beta-actin (C) in murine macrophage cells upon *igfbp3/scrambled*-siRNA and rGST-Otlip treatment. Black arrows denote the protein of interest. The cropped sections of these images are shown in main Figure 10.

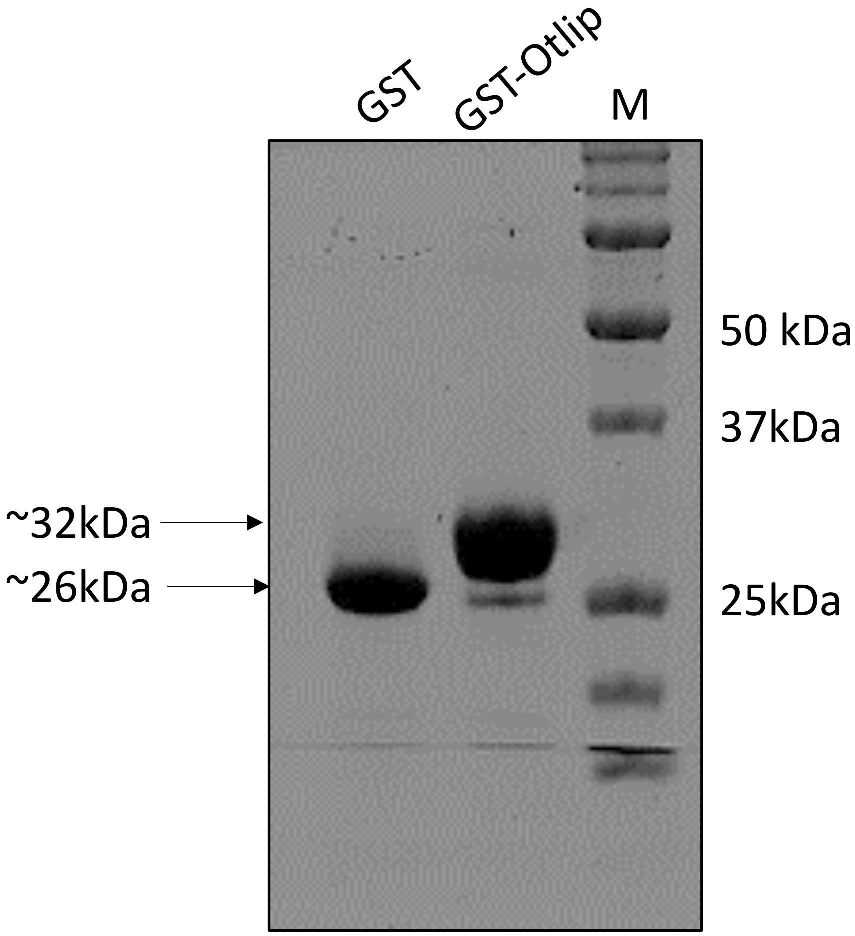

Supplementary Figure 1

Ray Bioseries -Mouse Cytokine Array C3 Layout(Catalog # AAM-CYT-3-2)

		A	B	C	D	E	F	G	H	I	J	K	L	M	N
Each antibody is spotted in duplicate vertically	1, 2	POS	POS	NEG	NEG	BLANK	Axl	BLC	CD30 Ligand	CD30	CD40	CRG-2	CTACK	CXCL-16	Eotaxin-1
	3, 4	Eotaxin -2	Fas-ligand	Fractalkine	GCSF	GMCSF	IFN- γ	IGFBP-3	IGFBP-5	IGFBP-6	IL-1α	IL-1β	IL-2	IL-3	IL-3Rβ
	5, 6	IL-4	IL-5	IL-6	IL-9	IL-10	IL-12p40/p70	IL-12p70	IL-13	IL-17A	CXCL-1	Leptin R	Leptin	LIX	L-Selectin
	7, 8	Ltn	MCP-1	MCP-5	MCSF	MIG	MIP-1α	MIP-1γ	MIP-2	MIP-3β	MIP-3α	PF4	P-selectin	RANTES	SCF
	9, 10	SDF-1α	TARC	I-309	TECK	TIMP-1	TNF-α	TNFRI	TNFRII	TPO	Vcam-1	VEGF-A	BLANK	BLANK	POS

Supplementary Figure 2

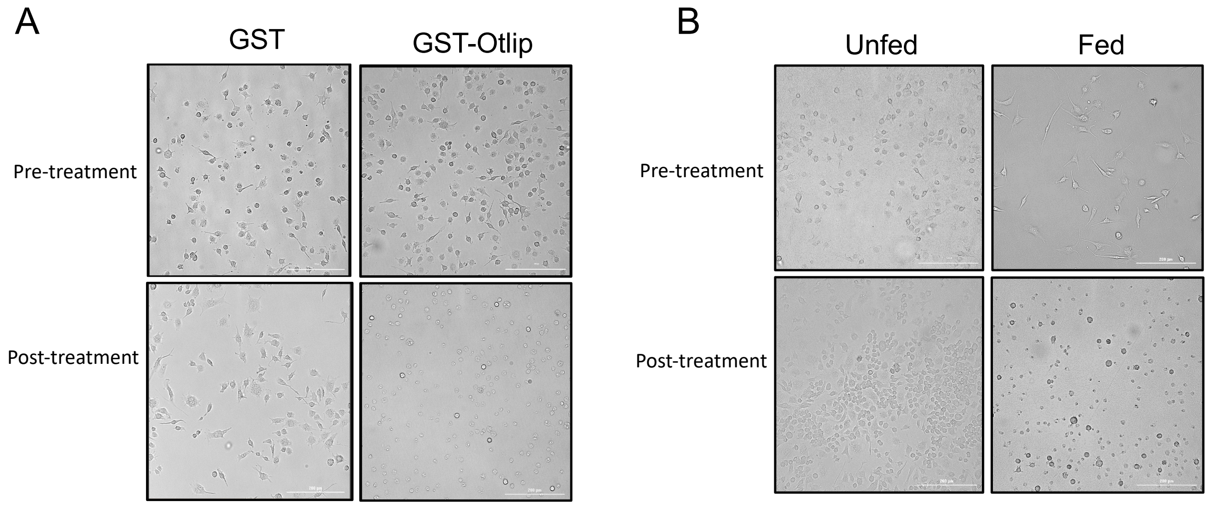

Supplementary Figure 3

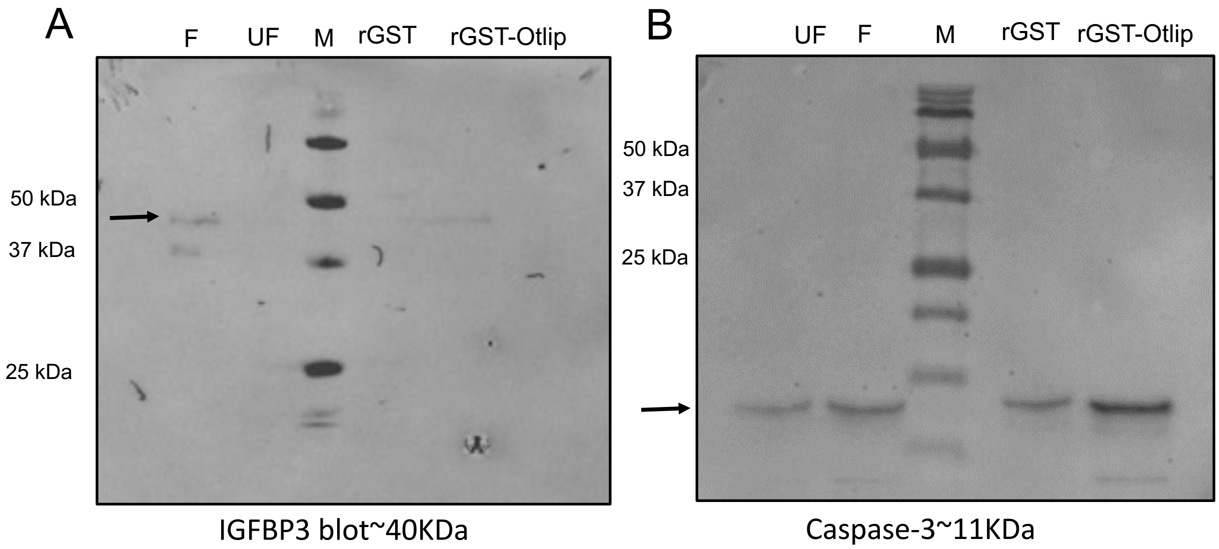

Supplementary Figure 4

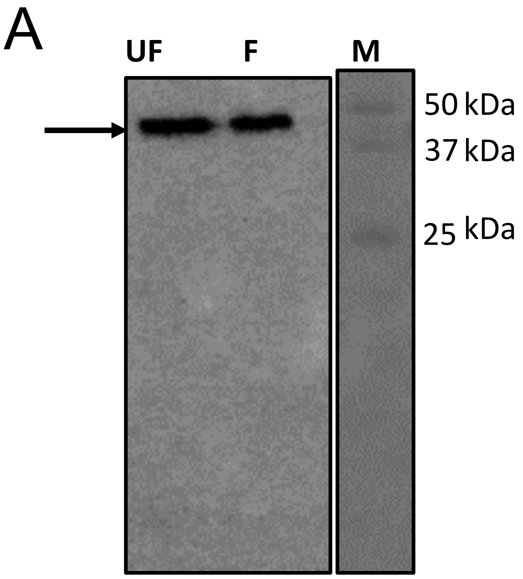

RAW Beta-Actin blot~40kDa

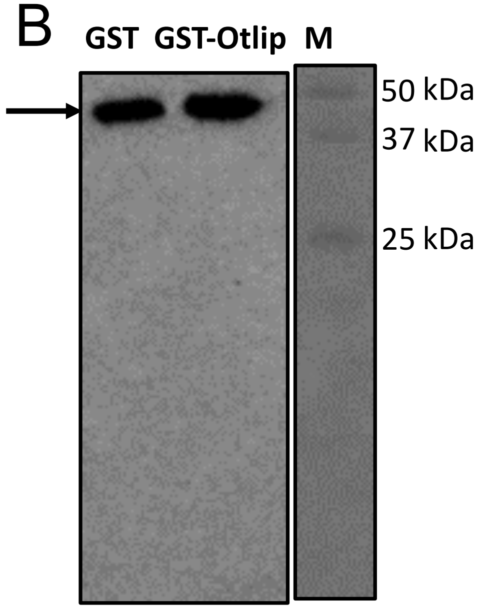

RAW Beta-Actin blot~40kDa

Supplementary Figure 5

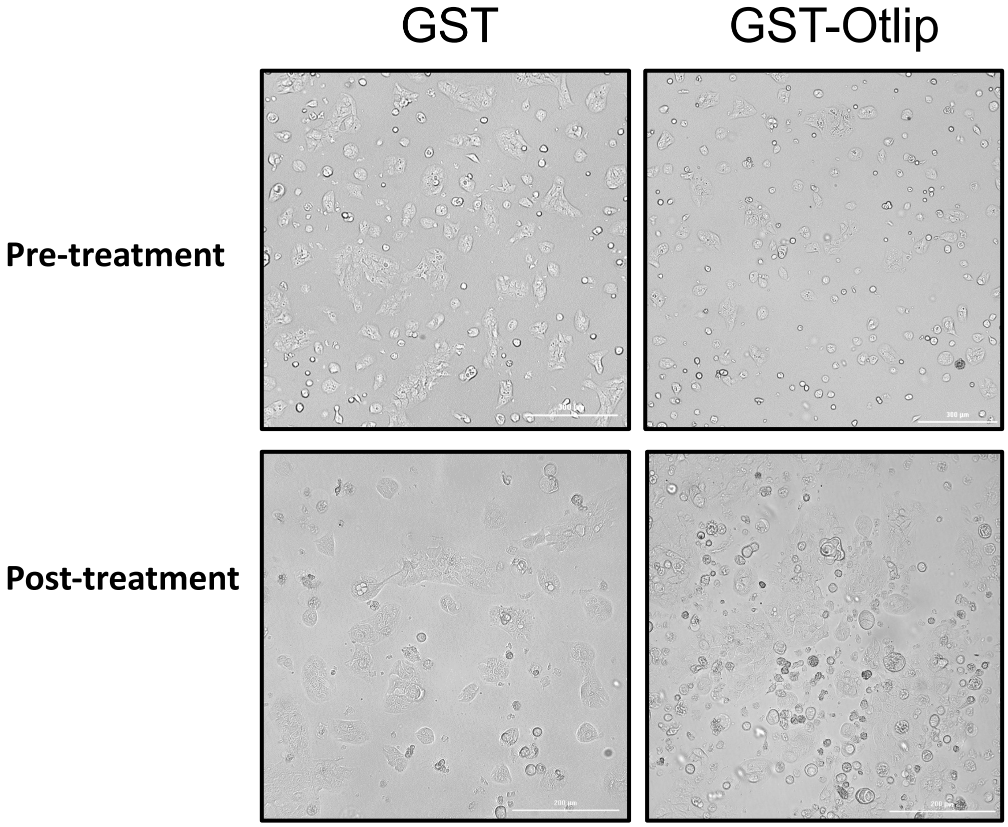

Supplementary Figure 6

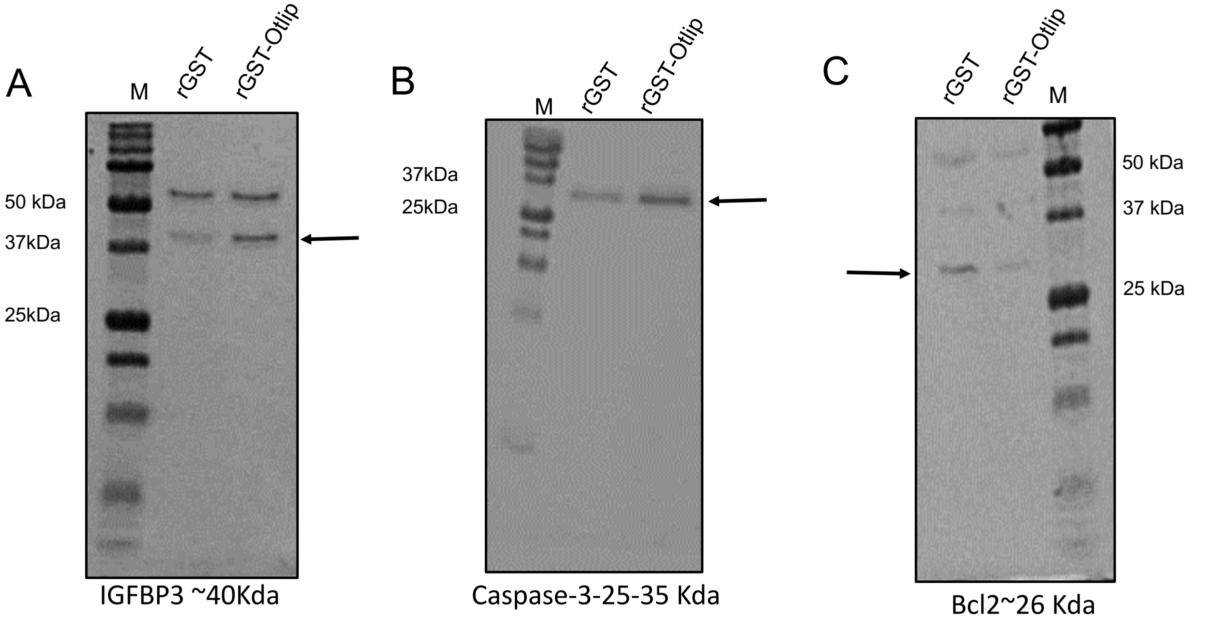

Supplementary Figure 7

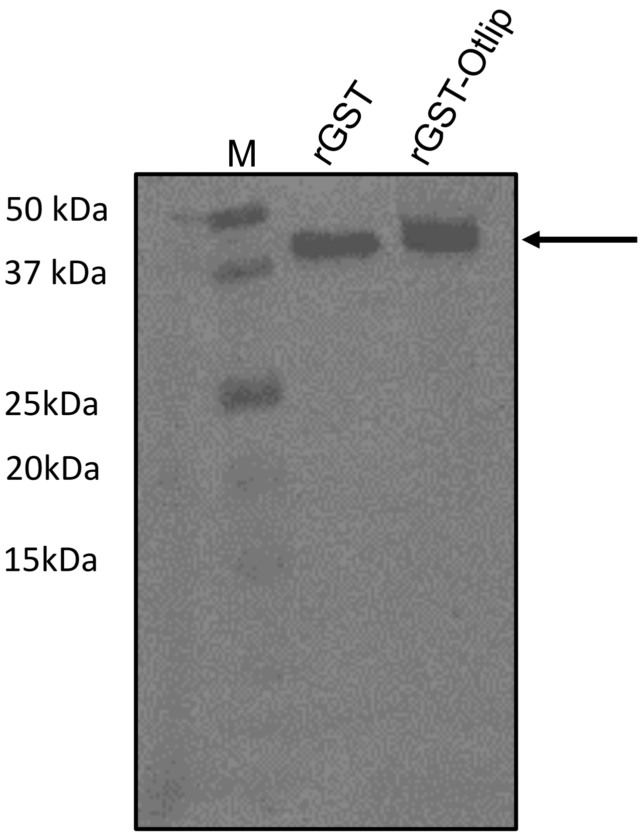

HaCat Beta-Actin blot~40kDa

Supplementary Figure 8

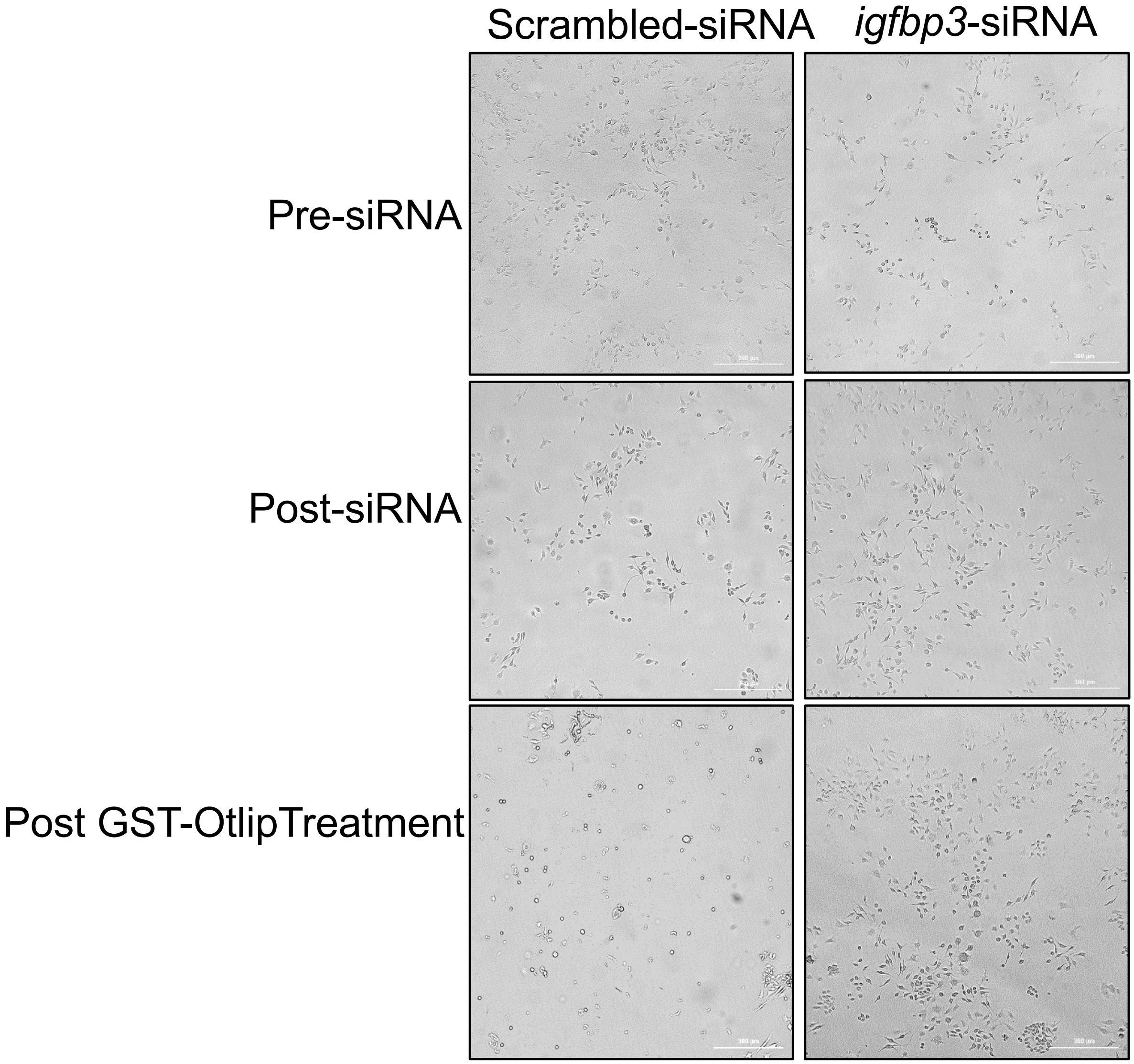

Supplementary Figure 9

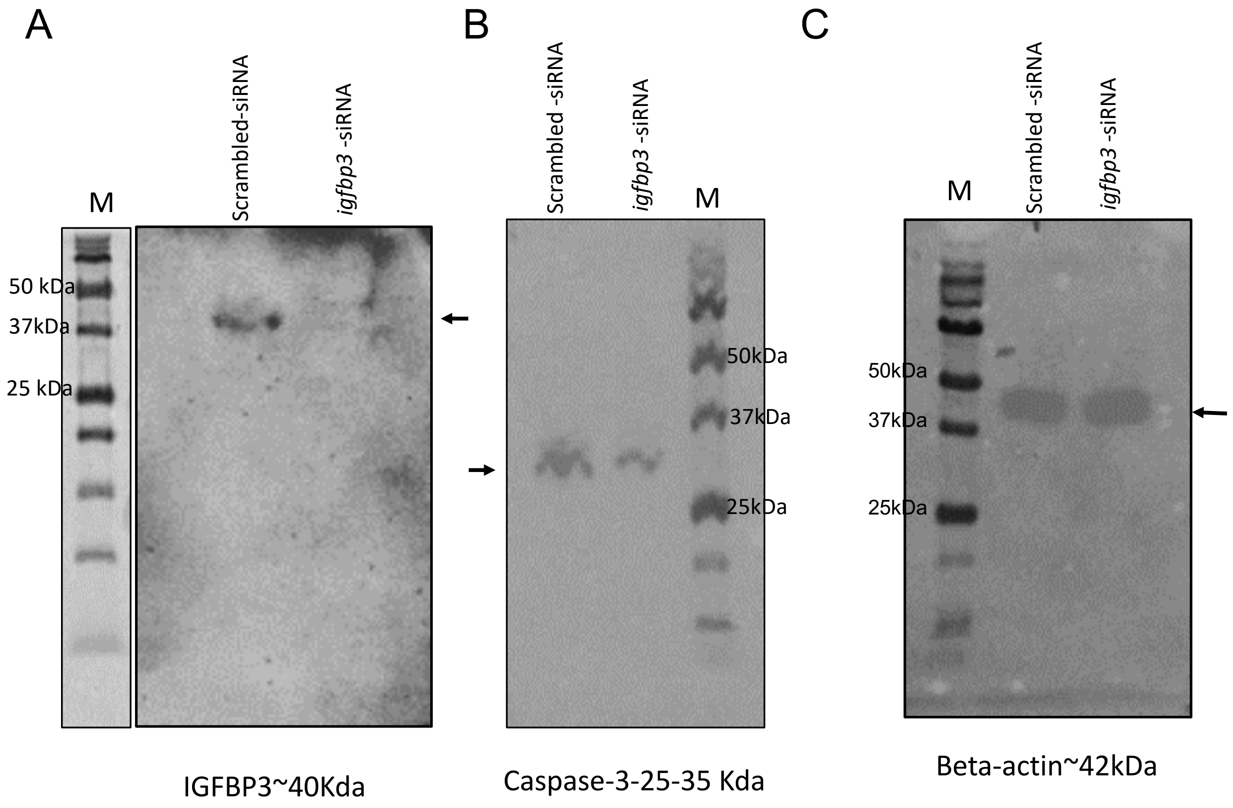

Supplementary Figure 10

Supplementary Table 1. Oligonucleotides used in this study

Sequence (5'-3')	Purpose
GGCAGCCTAAGCACCTACCT	Mice *igfbp3*, qRT-PCR
CCGCTTAGACTCGGAGGAGA	Mice *igfbp3*, qRT-PCR
CCCATGAATGAGACAGTCACA	mice *igfbp*-3R, qRT-PCR
CCCTGGAGTCCTTCCTGA	mice *igfbp*-3R, qRT-PCR
TGTGGCCTTCTTTGAGTTCG	mouse *bcl-2* primers, qRT-PCR
TCAGAGACAGCCAGGAGAAA	mouse *bcl-2* primers, qRT-PCR
CTACAGGGTTTCATCCAG	mouse *bax* primers, qRT-PCR
CCAGTTCATCTCCAATTCG	mouse *bax* primers, qRT-PCR
ATGCTCCGTGTCCATTGAGA	mouse *caspase-9*, qRT-PCR
AGTCACTGTCCAAGGTCCTG	mouse *caspase-9*, qRT-PCR
GAGCAGCTTTGTGTGTGTGA	mouse *caspase-3*, qRT-PCR
GGCAGGCCTGAATGATGAAG	mouse *caspase-3*, qRT-PCR
